# Identifying Clinical Managers’ Leadership Competencies: A Systematic Review and Cross-Frameworks Mapping Using the CLCF

**DOI:** 10.3390/healthcare14121720

**Published:** 2026-06-15

**Authors:** Ali Maashi, Julie Davies

**Affiliations:** 1 Global Business School for Health, University College London, 7 Sidings Street, Stratford, London E20 2AE, UK; 2 Brunel Business School, Brunel University of London, Kingston Lane, Uxbridge UB8 3PH, UK; julie.davies@brunel.ac.uk

**Keywords:** clinical leadership, leadership competency, healthcare management, systematic review, leadership perceptions, leadership challenges, leadership development

## Abstract

**Background/Objectives:** Effective clinical leadership is a critical driver of healthcare quality, patient safety, and organisational performance. However, evidence on the leadership competencies of healthcare professionals in formal management roles remains fragmented. It is dispersed across professional groups, healthcare contexts, and conceptual frameworks, limiting opportunities for synthesis and cumulative knowledge development. This systematic review examined three questions: how clinical managers perceive their leadership competency; what challenges they encounter in exercising leadership roles; and what development mechanisms the literature identifies. **Methods:** A systematic review was conducted following PRISMA 2020 guidelines and registered in PROSPERO (CRD420261305279). Four databases were searched: Ovid MEDLINE, CINAHL, EMCARE, and Web of Science from January 2010 to February 2026. Two reviewers independently screened studies; methodological quality was assessed using the Mixed Methods Appraisal Tool (MMAT). Reported competencies were mapped to the five domains of the Clinical Leadership Competency Framework (CLCF) using narrative integrative synthesis. **Results:** Forty-nine studies were included across quantitative, qualitative, and mixed-methods designs from 24 countries. Competencies in the Working with Others and Demonstrating Personal Qualities domains were reported as strengths across the largest number of included studies. Competencies in Managing Services, Improving Services, and Setting Direction were reported as areas of weakness or developmental need across multiple studies. Leadership challenges included inadequate preparation, role ambiguity, limited authority, and organisational constraints. Development needs spanned formal training, strategic competency building, mentoring, and sustained organisational support. **Conclusions:** Clinical leadership competency is unevenly distributed across CLCF domains. This pattern reflects not only individual developmental gaps but also the organisational and contextual conditions that shape how leadership is enacted in practice. The findings support a contextual-relational model of clinical leadership. Both individual capability and enabling organisational conditions must be addressed to strengthen leadership effectiveness across healthcare systems.

## 1. Introduction

Healthcare systems internationally are operating within increasingly demanding environments. Rising service utilisation, persistent workforce shortages, financial pressures, and ongoing organisational reform have collectively increased the complexity of coordinating care, managing performance, and sustaining quality improvement [1,2]. Within this environment, leadership has been widely recognised as a central influence on how healthcare organisations respond to systemic pressures and manage competing clinical and organisational priorities [3]. Analyses of healthcare quality have consistently indicated that shortcomings in patient safety and service performance are more often associated with organisational and leadership conditions than with isolated clinical errors [1,4]. The World Health Organisation estimates that unsafe care affects approximately 134 million patients annually in low- and middle-income countries, with a significant proportion of adverse events attributable to leadership deficiencies [5]. In high-income countries, studies suggest that up to 10% of hospitalised patients experience preventable harm linked to organisational and managerial conditions [6].

Leadership in healthcare has been examined as a complex social and organisational process. Healthcare organisations are characterised by strong professional groups, specialised expertise, and multiple centres of influence, all of which affect how authority and responsibility are distributed across organisational levels [7,8]. In such settings, leadership is enacted through interaction, negotiation, and professional credibility rather than through formal hierarchical authority alone [8]. These dynamics have important implications for service coordination, workforce engagement, and quality improvement [3,9]. The contribution of clinical leadership to these outcomes has been further evidenced through studies examining its role in aligning clinical practice with organisational objectives, particularly in relation to quality improvement, patient safety, and service effectiveness [3,9,10].

As the complexity of clinical leadership has become more apparent, scholarly attention has shifted toward competency-based approaches as a means of clarifying leadership expectations and supporting systematic development [3,9]. Within the management literature, competency is understood as a multidimensional construct integrating knowledge, skills, behaviours, and personal attributes required for effective performance in context [11]. This understanding recognises competency as both situational and developmental, shaped by the interaction between individual capability and contextual demands [11]. Competency-based frameworks have been advanced as practical tools for translating leadership concepts into clearly articulated and assessable capability domains [12].

In healthcare systems, such frameworks aim to articulate leadership capabilities relevant to multidisciplinary teamwork, service coordination, and system improvement [3]. In the United Kingdom, two policy-oriented frameworks were developed to guide this work across professional groups and organisational levels. The Medical Leadership Competency Framework (MLCF) was developed for doctors in leadership roles [13]. The Clinical Leadership Competency Framework (CLCF) extended this work to all regulated clinical professions, outlining five domains applicable to any healthcare professional in a leadership or management role: Demonstrating Personal Qualities, Working with Others, Managing Services, Improving Services, and Setting Direction [14].

The CLCF was selected as the organising framework for this review for three reasons. First, its scope explicitly encompasses all regulated clinical professions, from entry into formal training through to experienced practice, making it applicable to the full range of professional groups examined in this review [14]. Second, its five domains provide sufficient breadth to accommodate the competencies identified across the literature, while remaining specific enough to enable meaningful cross-study comparison. Third, the competencies captured within its five domains are conceptually consistent with those addressed in international frameworks, including the American Organisation for Nursing Leadership competency model [15], the National Center for Healthcare Leadership Health Leadership Competency Model [16], and the Management Competency Assessment Programme [17], despite differences in domain structure and professional scope [14]. The limitations of applying a UK-derived framework to international evidence are acknowledged in Section 4.6.

Despite the availability of these frameworks, evidence on clinical leadership competency remains conceptually and methodically fragmented. Studies have drawn on diverse theoretical perspectives, professional assumptions, and assessment approaches, resulting in variations in how leadership competency is defined and operationalised [18,19]. Research has tend to focus on specific professional groups, organisational context, or national health systems, limiting opportunities for synthesis and cross-contextual comparison [8,9]. Methodological diversity further complicates efforts to integrate findings in ways that support system-level understanding and evidence-informed leadership development [18].

This systematic review addresses this gap. The term ‘clinical managers’ is used here to encompass nurses, physicians, and allied health professionals, and other healthcare professionals with clinical backgrounds who occupy formal leadership, management, supervisory, administrative, or clearly recognised leadership responsibilities within healthcare settings. This population is consistent with the target scope of the CLCF, which applies to every clinician at all stages of their professional journey [14]. Three research questions guide the review: first, what leadership competencies do clinical managers perceive themselves to hold? Second, what are the contextual challenges that shape how those competencies are expressed in practice? Third, what development mechanisms does the literature identify as necessary to address the gaps that emerge? These questions are analytically connected: competency perceptions are shaped by the challenges encountered in practice, and both together inform what development is required. Examining them together, rather than in isolation, provides a more complete picture of clinical leadership competency in context. The findings are interpreted through three complementary theoretical frameworks: Mumford leadership Skills Model [20], Role Theory [21], and Situational Constraint Theory [22]. The findings are intended to inform both future research agendas and evidence-based leadership development initiatives across diverse healthcare systems.

## 2. Materials and Methods

### 2.1. Study Design and Reporting Standards

This systematic review was registered in the International Prospective Register of Systematic Reviews (PROSPERO; registration number CRD420261305279) on 9 February 2026, prior to completion of data extraction and manuscript submission. The search was updated to February 2026 as pre-specified in the PROSPERO protocol, with a documented end date of 28 February 2026. The review was conducted in accordance with the Preferred Reporting Items for Systematic Reviews and Meta-Analyses 2020 guidelines (PRISMA 2020) [23]. The SPIDER framework (Sample, Phenomenon of Interest, Design, Evaluation, and Research type) was used to operationalise the review questions and to inform the eligibility criteria and search strategy [24]. SPIDER was selected in preference to PICO because it accommodates qualitative and mixed-methods evidence and allows for flexible operationalisation of complex social phenomena such as leadership competency. PICO’s outcome-focused structure was considered less suitable for a review that includes qualitative and mixed-methods designs alongside quantitative studies.

### 2.2. Eligibility Criteria

Eligibility criteria were defined a priori and are summarised in Table 1. Studies were eligible for inclusion if they examined leadership competency (including competency perceptions, leadership challenges, or development needs) among healthcare professionals in formal or informal leadership and management roles. No restrictions were applied based on country of publication or healthcare setting. Only peer-reviewed articles published in English were included. Doctoral theses, dissertations, grey literature, and non-primary studies were excluded. The population examined in this review is consistent with the target scope of the CLCF, which applies to every clinician at all stages of their professional journey—from entry into formal training through to experienced practice [14]. All 49 included studies examined healthcare professionals in leadership or management roles, or preparing to assume such responsibilities, consistent with the pre-registered eligibility criteria.

### 2.3. Search Strategy

Four electronic databases were searched: Ovid MEDLINE, CINAHL, EMCARE, and Web of Science (a multidisciplinary citation index and search platform). The search was conducted from January 2010 to February 2026. The date restriction from 2010 was applied to capture contemporaneous evidence on clinical leadership competency frameworks, which gained prominence following the publication of the MLCF and CLCF in 2010–2011. A combination of keywords and database-specific subject headings was applied, with Boolean operators (AND and OR) used to combine search terms across titles and abstracts. The full search strategies for each database are provided in (Supplementary Table S1).

### 2.4. Study Selection

**Figure 1:**
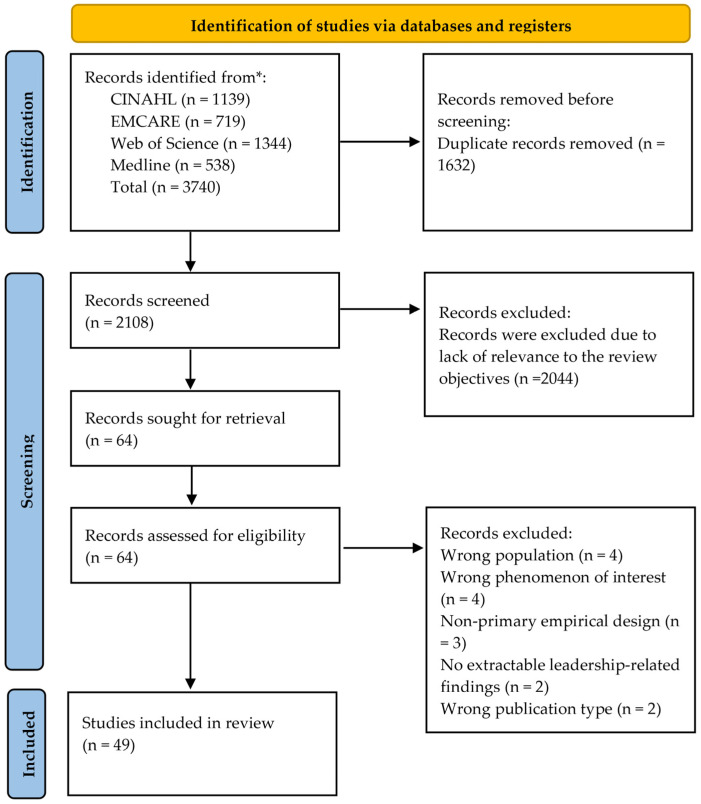
PRISMA flow diagram.

Records retrieved from the four databases were exported to Zotero for reference management. Duplicates were identified and removed using Zotero. The remaining records were imported into Rayyan for screening. Titles and abstracts were screened together against the predefined eligibility criteria by two reviewers (AM and JD) working independently. Disagreements were resolved through discussion until consensus was reached. Full-text articles of potentially eligible studies were then retrieved and assessed for eligibility. Reasons for exclusion at the full-text stage were documented and are reported in the PRISMA flow diagram (Figure 1).

### 2.5. Data Extraction

Data were extracted from all included studies using a predefined extraction form developed in Microsoft Excel. Extraction was performed independently by two reviewers (AM and JD). Discrepancies were resolved through discussion until consensus was reached. Extracted data included study characteristics: author, year, country, journal, setting, population, design, and sample size, as well as leadership-related components: assessment instruments or frameworks used, reported competency perceptions, identified challenges, and development needs.

### 2.6. Quality Appraisal

Methodological quality was assessed using the Mixed Methods Appraisal Tool (MMAT), version 2018 [25]. MMAT was selected because it enables appraisal of qualitative, quantitative, and mixed-methods studies within a single framework—a practical requirement given the heterogeneity of study designs included in this review. Following the initial screening questions, the relevant five-criterion set was applied according to each study’s design category. Each criterion was rated as “Yes”, “No”, or “Can’t tell”, consistent with MMAT guidance. No numerical scoring was applied. Quality appraisal was conducted independently by two reviewers (AM and JD), with discrepancies resolved through discussion. Results are presented in Supplementary Table S2.

The overall quality of the included studies was satisfactory. All 15 qualitative studies met all appraisal criteria. Among the 21 quantitative studies, five were rated “No” on one criterion each. Giri et al. [26], McGowan et al. [27], and Welch [28] were rated “No” on criterion 6 (risk of non-response bias), reflecting limitations in response rate reporting. Mrayyan [29] and Pokhrel et al. [30] were rated “No” on criterion 4 (representativeness of the sample), indicating potential sampling limitations. Among the 13 mixed-methods studies, three demonstrated more notable limitations. Gulati [31] was rated “No” on four criteria, relating to the rationale for the mixed-methods design, integration of components, interpretation of outputs, and adherence to methodological quality standards. Isibor et al. [32] was rated “No” on three criteria concerning the integration and interpretation of qualitative and quantitative components. Liou et al. [33] was rated “No” on criterion 6, relating to the handling of divergences between quantitative and qualitative results. These limitations were considered in the interpretation of findings from the affected studies. Notwithstanding individual variation in study quality, the overall pattern of findings was consistent across higher- and lower-quality studies, supporting confidence in the principal conclusions of this review.

### 2.7. Data Synthesis

The data were synthesised using narrative integrative synthesis, an approach appropriate for reviews incorporating qualitative, quantitative, and mixed-methods evidence [34,35]. Although meta-analysis is technically feasible for homogeneous quantitative datasets, and meta-synthesis for qualitative evidence, neither was appropriate here. The 49 included studies employed heterogeneous designs, diverse leadership frameworks, and non-comparable outcome measures. No universal measure was consistently reported across studies to permit quantitative pooling. Narrative synthesis was therefore the most methodologically appropriate approach, consistent with the pre-registered PROSPERO protocol.

The synthesis followed three stages. The first stage involved contextual grounding: the descriptive characteristics of the included studies (settings, professional groups, and frameworks) were summarised to provide a transparent foundation for interpreting findings. The second stage involved systematic extraction and mapping: data were extracted in relation to the three review questions and mapped to the five CLCF domains. For studies without an explicit framework, competencies were inductively mapped to the most relevant CLCF domain based on functional alignment. The full mapping process, including decisions made for each included study, is documented in (Supplementary Table S4). The third stage involved coding and consistency checking: competencies were independently coded by two reviewers (AM and JD) against the CLCF domains. Inter-rater agreement was established through structured discussion meetings held throughout the review process. Disagreements were resolved by reference to the predefined CLCF domain definitions until consensus was reached across all 49 studies. Where competencies were ambiguous or did not map clearly to a single domain, these were discussed in detail and resolved through consensus, with all mapping decisions documented in Supplementary Table S4.

Quantitative findings were summarised descriptively. Qualitative insights were synthesised to preserve contextual meaning. Patterns were examined across professional groups and healthcare settings. The influence of study quality on the findings was considered throughout: where MMAT appraisal identified methodological limitations in individual studies, these were noted in the interpretation of relevant findings and are discussed further in Section 4.6.

## 3. Results

### 3.1. Selected Studies

A total of 3740 records were identified through database searches across four electronic databases and exported to Zotero for reference management. After removal of 1632 duplicate records, 2108 unique articles remained for screening. Titles and abstracts of these records were screened using Rayyan software against the predefined inclusion and exclusion criteria. Following this initial screening, 2044 records were excluded due to lack of relevance to the review objectives. The remaining 64 records were sought for retrieval and assessed for eligibility. Of these, 15 were excluded after full-text review: wrong population (*n* = 4), wrong phenomenon of interest (*n* = 4), non-primary empirical design (*n* = 3), no extractable leadership-related findings (*n* = 2), and wrong publication type (*n* = 2). As a result, 49 primary studies were included in the final systematic review. The selection process is illustrated in the PRISMA flow diagram (Figure 1).

### 3.2. Characteristics of Included Studies

**Figure 2:**
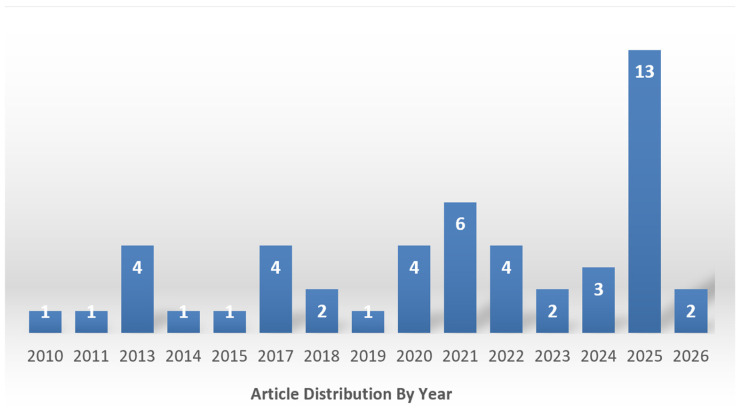
Article Distribution by Year. Note: 2026 reflects publications identified up to the search end date of 28 February 2026 and does not represent a full calendar year.

Forty-nine primary studies met the inclusion criteria. Studies were published between 2010 and 2026, reflecting growing scholarly interest in clinical leadership competency over the past decade. As illustrated in (Figure 2), publication activity increased notably after 2015, with a peak observed in more recent years.

**Figure 3:**
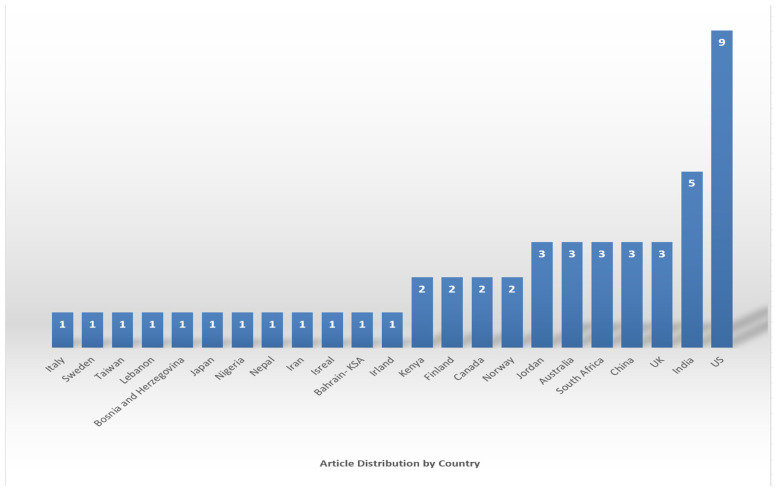
Article Distribution by Country.

Figure 3 illustrates the geographical distribution of included studies across 24 countries. The United States contributed the largest number of studies (*n* = 9), followed by India (*n* = 5). Five countries each contributed three studies: Jordan, Australia, South Africa, China, and the United Kingdom. Four countries contributed two studies each: Kenya, Finland, Canada, and Norway. The remaining 12 countries each contributed one study: Italy, Sweden, Taiwan, Lebanon, Bosnia and Herzegovina, Japan, Nigeria, Nepal, Iran, Israel, Bahrain and Saudi Arabia, and Ireland. The concentration of evidence within high-income countries, particularly the United States, may limit the transferability of findings to lower-resource healthcare settings and introduces potential framework bias, given that several included studies drew on frameworks developed within Western health systems. This limitation is addressed further in Section 4.6.

Of the 49 included studies, 21 adopted a quantitative design [26,27,28,29,30,36,37,38,39,40,41,42,43,44,45,46,47,48,49,50,51], 15 employed qualitative methodology [52,53,54,55,56,57,58,59,60,61,62,63,64,65,66], and 13 utilised mixed-methods approaches [31,32,33,67,68,69,70,71,72,73,74,75,76]. This diversity supports the use of narrative integrative synthesis and reflects the multifaceted nature of clinical leadership research across different professional groups and healthcare contexts.

Study populations included nurses, physicians, and allied health professionals in formal or informal leadership and management roles, including nurse managers, physician managers, clinical directors, and senior healthcare professionals. Sample sizes ranged from fewer than 30 participants in small qualitative studies to several hundred respondents in large-scale quantitative surveys.

Healthcare settings included public and private hospitals, community health services, primary healthcare centres, long-term care facilities, and national health system contexts. Leadership competency was assessed using a range of instruments: self-assessment tools, surveys, semi-structured interviews, and mixed-methods approaches. Several studies employed established frameworks including the MLCF, CLCF, MCAP, and AONE model. A substantial number did not employ a formal framework and instead explored competencies through context-specific constructs and empirical findings.

A detailed summary of study characteristics: including country, setting, population, design, sample size, frameworks used, key findings, challenges, and development needs, is presented in (Supplementary Table S3).

### 3.3. Perceptions of Leadership Competency Among Clinical Managers

The 49 included studies examined leadership competency perceptions among clinical managers across diverse healthcare settings and professional groups. Reported competencies were mapped to the five domains of the CLCF [14], as documented in Supplementary Table S4. A summary of the distribution of perceived competency across domains is presented in Table 2.

The findings reveal a consistent pattern across the included studies. Competencies in the interpersonal and personal domains were reported as areas of relative strength, while competencies in the strategic, managerial, and service improvement domains were reported as areas of relative weakness or developmental need. This pattern was observed across diverse professional groups, healthcare settings, and national contexts.

Competencies within the **Working with Others** domain were reported as strengths across the largest number of included studies. Communication, teamwork, and relationship management were documented consistently across professional groups and national contexts [27,33,37,51,67,68,72]. Where limitations were identified, they clustered in the more advanced interpersonal competencies, conflict management and influencing others, rather than in basic communication [28,48,68,77]. This distinction matters: the strength is in day-to-day relational function, not in the exercise of interpersonal influence under organisational pressure.

The **Demonstrating Personal Qualities** domain was similarly reported as well-developed. Integrity, professionalism, and self-management were documented across multiple studies [27,32,46]. Ethical leadership qualities (including compassion and role modelling) were highlighted across studies examining diverse professional groups [26,55,77]. What varied was not the presence of these qualities but their stability under pressure; indeed, several studies identified gaps in autonomy and leadership maturity [28,52,53,67].

The **Managing Services** domain presents a different picture. Basic operational functions, specifically, planning and execution, were reported as adequate in some studies [45,67,68]. But the advanced managerial competencies, including financial management, human resource management, and resource allocation, were reported as underdeveloped across a substantially larger body of evidence [29,37,38,41,42,48,77]. The gap is not in operational competency. It is in the competencies required to manage services as a system.

The **Improving Services** domain reveals a similar structure. Engagement with quality activities and performance monitoring was documented in some studies [33,57,72]. But innovation, change management, and service transformation capabilities were reported as limited across a larger body of evidence [28,38,42,43,50,67,68,76].

The **Setting Direction** domain produced the most consistent finding across the evidence base. Basic decision-making at the operational level was documented in some studies [32,44,63,66,71]. Strategic thinking, vision setting, and policy development were reported as lacking across the greatest number of included studies and the widest range of contexts [26,38,41,45,48,50,68,76,77]. This is the domain furthest from the operational core of clinical management, and it is consequently the one that requires the most from the organisational environment.

These findings indicate that clinical managers demonstrate stronger capabilities in interpersonal and personal leadership domains than in strategic, managerial, and service improvement domains. This imbalance is not adequately explained by individual developmental gaps alone. As the following section demonstrates, it is closely linked to the organisational and contextual conditions in which leadership is exercised.

### 3.4. Leadership Challenges

The challenges reported across the 49 included studies do not describe a problem of individual capability. They describe a problem of enabling conditions. Organisational constraints such as limited authority, bureaucratic cultures, and resource shortages restrict how leadership is expressed in practice. What has been labelled a competency deficit in much of the quantitative literature is, in many cases, a leadership enactment deficit produced by environmental conditions that training cannot address. A summary of the major challenge categories is presented in Table 3.

Training and preparation challenges were reported across the largest number of included studies and the widest range of national contexts, indicating that inadequate preparation for leadership roles is a cross-contextual rather than context-specific concern. Clinical managers reported entering leadership positions without formal training or structured development [39,42,54,67,77]. Several studies also highlighted the absence of structured development pathways, leaving managers dependent on experiential learning [55,57,70]. The consistency of this finding across study designs and national contexts gives it the character of a structural condition rather than an individual circumstance.

Organisational and structural challenges were reported across studies from diverse settings. Limited decision-making authority, unclear governance structures, and bureaucratic constraints were the dominant forms [61,67,76]. Performance pressures, financial constraints, and policy limitations further restricted leadership effectiveness in some contexts [50,74]. Resource constraints, including insufficient staffing and budget limitations, were additional barriers [30,65]. What these studies collectively describe is not a set of discrete problems but a structural condition in which leadership authority is formally assigned but practically constrained.

Role-related challenges were reported predominantly in qualitative studies, representing a methodological pattern suggesting these challenges are better captured through in-depth narrative inquiry than through survey instruments. Clinical managers described tension between clinical and managerial responsibilities, unclear role expectations, and difficulties navigating professional hierarchies [59,66,68]. This role conflict was compounded by expectations to manage peers and balance competing priorities [54,62]. The dual-role tension is not a personal failing. It is the predictable consequence of placing a clinician in a management role without resolving the structural and cultural conditions that make the two roles conflict.

Workload pressure and time constraints were reported consistently. Heavy operational demands, staff shortages, and competing priorities depleted the capacity of clinical managers to engage in strategic leadership activities [44,47,61]. The mechanism here is direct: when operational demand consumes available capacity, strategic competency is the first casualty.

Relational and professional challenges included conflict management difficulties, limited support from senior management, and resistance to change [27,32,53]. Managing interpersonal dynamics and multidisciplinary team boundaries was reported as particularly demanding in complex healthcare environments.

Socio-cultural and contextual challenges including, hierarchical cultures, political pressures, and gender-related barriers, were identified in fewer studies [60,73]. Their presence in the evidence base, even at lower frequency, points to a dimension of clinical leadership constraint that the quantitative literature has largely failed to capture.

These findings, taken together, describe a consistent structural logic. Clinical managers who lack adequate preparation encounter organisations that restrict their authority, deplete their capacity, and contest their legitimacy. The competency distribution reported in Section 3.3 is the predictable output of this logic. It is not what these managers cannot do. It is what their environments do not permit them to do.

### 3.5. Leadership Development Needs

The development needs reported across the included studies have a specific structure that a surface reading of the evidence can obscure. They are not randomly distributed. They cluster in precisely the domains where competency is weakest (specifically Managing Services, Improving Services, and Setting Direction), and they reflect the same environmental logic that the challenge data in Section 3.4 describe. Understanding the structure of these needs requires attention not only to what is needed but to how the need was identified. A small number of studies captured needs directly through participant self-assessment [39,51]. The majority reported needs through researcher inference from observed competency gaps [41,57,67,70]. A further set identified needs through qualitative accounts of lived leadership experience [54,62,64]. A summary of the major development areas is presented in Table 4.

The most consistently identified need was formal leadership training prior to role assumption. It was reported across quantitative surveys, qualitative interviews, and mixed-methods studies, and it was evident across the widest range of national contexts and professional groups. Clinical managers reported entering leadership roles without adequate preparation [39,41,45,57,66,67,70]. The consistency is not surprising given what the challenge data showed; What is notable, however, is that this pattern persists across contexts where structural conditions are otherwise very different, thereby suggesting that the absence of pre-role preparation is a universal feature of how clinical leadership is currently organised.

Strategic and managerial competency development was the second most consistently identified need, carrying the most specific analytical implications. Financial management, human resource management, policy literacy, and system-level knowledge were the competencies most often identified as requiring development [30,33,37,38,42,67,68,71]. This need was particularly prominent in studies examining physicians and senior managers, highlighting a specificity that generic training programmes do not address.

Interpersonal and communication competency development was reported across both quantitative instruments and qualitative practice accounts. Communication, conflict management, teamwork, and relationship management were identified as ongoing requirements rather than one-time training needs [32,47,51,53,56,72].

Mentoring, coaching, and peer learning were identified as development mechanisms rather than competency domains. The evidence does not show that clinical managers lack mentoring skills. It shows that they lack access to structured mentoring relationships [59,62,64,68,76]. Their value lies not in the content they deliver but in the conditions they create.

Continuing professional development was identified as a sustained infrastructure requirement rather than a discrete intervention [63,65,74]. One-off training programmes cannot address the depth of the developmental gap. The gap requires a system, not an intervention.

Broader organisational support was identified as a prerequisite for development rather than a supplement to it [60,61,76]. Protected time, governance reform, and enabling environments are the structural conditions without which individual development investment cannot produce system-level improvement.

The structure of the development needs mirrors the structure of the competency gaps. The most consistently unmet needs are not in domains where clinical managers are already strong. They are in the domains where the gap between current capability and role demand is greatest. Addressing these gaps requires not only targeted development programmes but also the organisational conditions that make sustained development possible.

## 4. Discussion

### 4.1. Overview of Key Findings

The findings of this systematic review produce a pattern that is both consistent and analytically significant. Forty-nine studies, conducted across 24 countries and spanning quantitative, qualitative, and mixed-methods designs, converge on the same distribution of leadership competency. Interpersonal and personal competencies are stronger. Strategic, managerial, and service improvement competencies are weaker. The pattern holds across nurses, physicians, and allied health professionals. It holds across high-income and lower-resource settings. It holds across studies that used formal frameworks and studies that did not. A finding that replicates at this scale, across this level of methodological and contextual diversity, is not incidental. It requires explanation.

This review offers that explanation through three analytically connected dimensions. The first concerns what clinical managers perceive themselves to be capable of. The second concerns the contextual conditions that shape how those competencies are expressed in practice. The third concerns what development is required to address the gap between capability and enactment. These three dimensions are not independent lines of inquiry. Competency perceptions are shaped by the conditions in which leadership is exercised. Both together determine what development is actually required. A review that examined any one of these dimensions in isolation would produce findings that are technically accurate but analytically incomplete.

To our knowledge, this is the first systematic review to examine all three dimensions together, within a unified analytical framework mapped to the CLCF, across 49 studies spanning 24 countries and multiple professional groups.

### 4.2. Fragmentation of the Leadership Competency Framework

The evidence base examined in this review is characterised by substantial framework heterogeneity. Of the 49 included studies, 18 employed no formal competency framework. A further 19 used profession-specific or context-specific instruments, including the AONE model [28,52], the MCAP framework [30,42,43,58], the NCHL model [45], and various locally developed tools. Five studies explicitly employed frameworks from the CLCF/MLCF family. This fragmentation is not a reporting inconsistency. It reflects a genuine absence of consensus about what clinical leadership competency is, how it should be defined, and how it should be measured. That absence has consequences: without a common analytical language, findings accumulate but do not build.

The selection of the CLCF as the organising framework for this review rests on three considerations that the evidence now supports. First, the CLCF was developed as a direct evolution of the MLCF, extending its application from doctors to all regulated clinical professions while retaining the same five-domain structure [14]. Four of the 49 included studies employed the MLCF explicitly [41,67,68,77], and one employed the CLCF directly [73]. Second, the framework’s applicability extends beyond its NHS origins. Gulati et al. [41,77] employed the MLCF across two independent studies in India. Shikama et al. [73] applied the CLCF in Japan through a culturally adapted Delphi process, ultimately producing an 84-item version that retained the original domain structure while reflecting Japanese sociocultural values. Cultural adaptation was required. Wholesale replacement was not. Third, of the 43 remaining studies, the competencies reported mapped naturally to CLCF domains through inductive functional analysis, as documented in Supplementary Table S4. The framework was not imposed on evidence that resisted it. It accommodated evidence that had no common structure.

The limitation of applying a UK-derived framework to international evidence is acknowledged. No competing framework, however, offers comparable breadth across clinical professions, comparable international validation, or comparable domain coverage across the diversity of professional groups represented in this review. The CLCF is not the only possible organising lens. It is the most defensible one available for this purpose.

The fragmentation itself has a direct implication for interpretation. When 18 studies report findings without any framework and 19 others use instruments designed for specific professional groups, synthesis necessarily involves researcher judgement in mapping reported competencies to a common structure. That judgement was exercised through independent coding, structured discussion, and consensus, following the process described in Section 2.7 and documented in Supplementary Table S4. The limitation is real, but the analytical alternative, which would involve abandoning cross-study comparison entirely, yields a body of literature that remains rich in individual detail but limited in its cumulative scientific value.

### 4.3. Distribution of Competencies Across CLCF Domains

The distribution of perceived competency across the five CLCF domains is not uniform and this the non-uniformity is not random. The task of this section is not to restate the pattern documented in the Results but to explain it.

Before offering that explanation, one methodological qualification is necessary. The prominence of interpersonal competencies in the synthesis may partly reflect measurement bias. Studies using instruments designed to assess interpersonal leadership will naturally yield stronger evidence of interpersonal competency, not necessarily because it is more developed, but because it is more measured. Future research employing comprehensive multi-domain instruments would be needed to disentangle this from genuine developmental differences.

What the synthesis can say with confidence is this: the three-domain weakness is documented consistently across diverse professional groups and national contexts [26,30,42,48]. Interpersonal competency strengths were documented across multiple studies [32,33,64,71,72]. Individual-level attributes were widely reported [39,74,75,77]. Strategic and service direction deficits were documented consistently [26,30,33,38]. Managing Services limitations were similarly prominent [37,45,48,77]. Improvement-related limitations were consistent across studies [38,43,57,74]. A pattern that replicates at this level of consistency is not adequately explained by measurement artefact alone. Something structural is producing it, and the following section provides a theoretically grounded account of what that structure is.

### 4.4. Contextual and Theoretical Interpretation of Leadership Competency Distribution

The pattern documented across the 49 included studies requires a theoretical account. Three complementary frameworks are drawn upon here, not to establish causality, which the review design does not permit, but to provide an analytical account of why this pattern is consistent with what theory would predict.

The first is Mumford et al.’s [20] Leadership Skills Model, which proposes that leadership effectiveness involves hierarchically organised skill categories whose expression is contingent on environmental conditions. The findings of this review are consistent with this framework. Social judgement skills comprising communication, teamwork, and relationship management, are reported as strengths because they are embedded in day-to-day leadership practice and are reinforced by the daily operational demands of clinical management roles. Complex problem-solving and system-level capabilities, by contrast, require enabling conditions, such as decision-making authority, strategic access, and protected cognitive space, that the contextual challenge data show to be routinely constrained. While quantitative studies primarily identified differences in perceived competency [45,48,49,77], qualitative studies provide insight into the organisational and professional conditions shaping leadership practice [54,59,60,61]. Mixed-methods studies further bridge these perspectives by linking competency patterns with contextual challenges [32,67,68,73,74].

The second is Role Theory [21], which proposes that role conflict generates predictable patterns of performance limitation. The clinical manager occupies a role that combines two distinct institutional logics, namely clinical professionalism and organisational management, whose demands frequently conflict. Leadership roles are consistently described as being embedded within operationally demanding environments characterised by workload pressures, time constraints, and resource limitations [61,68,74,75]. Under these conditions, leaders prioritise coordination and interpersonal engagement, which are activities that maintain continuity of care, over longer-term strategic engagement [3]. Role Theory would predict that competencies requiring unambiguous managerial authority, such as Setting Direction, Managing Services, are most constrained by this conflict, while competencies anchored in the clinical professional identity are least constrained. Several studies identified contextual constraints such as limited decision-making authority, bureaucratic structures, and restricted involvement in organisational strategy [30,36,68,76]. These factors limit opportunities for strategic engagement and are consistent with what Role Theory would predict. The role structure, in other words, produces the competency distribution. Training cannot resolve a conflict that is structural in origin [7].

The third is Organisational Constraint Theory [22]. Multiple studies reported challenges including inadequate preparation for leadership roles, role ambiguity, and tensions between clinical and managerial responsibilities [41,43,54,66]. These conditions affect the development and application of competencies related to service management and operational oversight. Resource limitations, organisational resistance, and competing priorities reduce engagement with quality improvement and change initiatives [38,42,57,74]. In such contexts, leaders prioritise maintaining existing service delivery over initiating improvement efforts, reflecting a broader tension within healthcare systems where demands for operational continuity limit the capacity for innovation [34]. The influence of socio-cultural and professional dynamics adds a further layer: gendered expectations, informal power structures, and professional hierarchies shape access to leadership opportunities and influence how roles are enacted [60].

Together, these three frameworks support an interpretation this review terms contextual-relational: that clinical leadership competency is not adequately understood as a fixed property of an individual but as a function of the interaction between individual capability, role structure, and organisational environment. This interpretation is proposed on the basis of the pattern of associations documented across the 49 included studies. It is not established by this review as a causal claim. Whether and how these associations operate in specific organisational and cultural contexts, as well as what mechanisms produce them, are empirical questions that primary qualitative research in defined healthcare settings is better positioned to address.

### 4.5. Implication for Leadership Theory and Practice

The findings carry implications at three levels, namely individual, organisational, and system, that do not operate independently. An intervention at one level without corresponding change at the others is unlikely to produce sustained improvement in clinical leadership effectiveness. That is not a general claim about leadership development. It is a specific inference from the pattern this review documents.

**At the individual level**, the most consistently identified need is structured preparation before role assumption. Clinical managers are currently entering leadership roles without the formal training, competency-based preparation, or structured development pathways that the evidence base shows they require [39,41,45,57,66,67,70]. The development gap is not uniform across domains. Strategic, financial, and system-level competencies, specifically those corresponding to the Managing Services, Improving Services, and Setting Direction domains, are the ones most consistently underdeveloped and the ones least addressed by generic leadership programmes.

**At the organisational level**, the challenge data establish that individual development investment is necessary but insufficient. The most consistently reported organisational constraints, including limited decision-making authority, heavy workload, and unclear governance structures, restrict the expression of strategic and managerial competencies regardless of individual capability level. The enabling conditions most consistently absent, such as protected time for leadership work, clarity of role authority, and institutional cultures that legitimise strategic engagement, are prerequisites for development investment to produce outcomes in the domains of greatest need.

**At the system level**, the contextual-relational interpretation proposed in Section 4.4 suggests that the interpersonal-strategic competency gap documented across 49 studies is not the product of individual developmental failure but of how clinical leadership roles are currently structured internationally. Addressing this requires workforce planning that builds leadership preparation into clinical career pathways, governance reform that clarifies and protects managerial authority, and policy frameworks that recognise enabling conditions as a prerequisite for leadership effectiveness.

Quality appraisal using MMAT indicated that the overall pattern of findings remained consistent across higher- and lower-quality studies. Studies rated lower on methodological rigour contributed findings consistent with those from stronger designs. This consistency strengthens the evidential basis for the implications described above, without eliminating the limitations that individual study quality imposes, which are limitations addressed in Section 4.6.

The contextual-relational model proposed in this review represents a specific theoretical contribution. It advances on the competency-deficit interpretation, which positions the three-domain weakness as a training problem, by demonstrating that the pattern is consistent with an enabling conditions explanation that training alone cannot address. Leadership competency should not be treated as an individual attribute to be developed and deployed independently of context. It is a contextually embedded capability whose expression depends on the interaction between what individuals can do and what their environments permit them to do.

### 4.6. Strengths and Limitations

This review has several methodological strengths. It is the first systematic review to integrate three analytically connected dimensions of clinical leadership competency, comprising perceived competencies, contextual challenges, and development needs, within a unified CLCF-based framework across 49 studies from 24 countries. The use of a pre-registered protocol (PROSPERO; CRD420261305279), PRISMA 2020 guidelines, and MMAT quality appraisal provides a transparent and reproducible methodological foundation across quantitative, qualitative, and mixed-methods designs.

However, several limitations must be acknowledged. First, restriction to English-language publications introduces language bias, potentially excluding relevant evidence from non-English-speaking contexts. Second, grey literature was not searched, which may have missed policy-relevant evidence on leadership competency frameworks and development practices. Third, publication bias cannot be excluded, as studies reporting significant findings are more likely to be published. Fourth, the heterogeneity of study designs, populations, and measurement approaches limits direct comparability and precludes meta-analytic synthesis. Fifth, most included studies relied on self-reported competency measures, subject to social desirability bias. Sixth, the use of the CLCF as a mapping framework introduces potential reviewer subjectivity; this was mitigated through independent coding and structured consensus documented in Supplementary Table S4. Seventh, the evidence base is concentrated in high-income countries, particularly the United States and United Kingdom, limiting transferability to lower-resource settings. Eighth, PROSPERO registration was completed concurrent with early data extraction, which is acknowledged as a timeline limitation. Ninth, the restriction to two reviewers without formal inter-rater reliability calculation is noted, although consensus was reached in all cases through structured discussion. Tenth, backward and forward citation searching was not conducted, which may have resulted in the exclusion of relevant studies not identified through database searching alone.

Regarding study quality, all 15 qualitative studies met all MMAT criteria. Five quantitative studies were rated “No” on one criterion each, primarily relating to response rate reporting and sample representativeness [26,27,28,29,30]. Three mixed-methods studies demonstrated more notable limitations: Gulati [31] on four criteria, Isibor et al. [32] on three, and Liou et al. [33] on one. Despite this variation, the overall pattern of findings remained consistent across higher- and lower-quality studies, supporting confidence in the principal conclusions.

## 5. Conclusions

Clinical leadership competency is not uniformly distributed across the five CLCF domains. The pattern documented across 49 studies from 24 countries is consistent: competencies in the interpersonal and personal domains are reported as strengths, while competencies in the strategic, managerial, and service improvement domains are reported as areas of weakness or developmental need. This pattern holds across professional groups, study designs, and national contexts. Its consistency at that scale requires explanation beyond individual developmental deficit.

The contextual-relational interpretation proposed in this review offers that explanation. The three-domain weakness in Managing Services, Improving Services, and Setting Direction is consistent with what Mumford et al.’s [20] Leadership Skills Model, Role Theory [21], and Organisational Constraint Theory [22] would predict when enabling conditions are systematically absent. Clinical managers who enter leadership roles without adequate preparation encounter organisations that constrain their authority, deplete their capacity, and contest their legitimacy. The competency distribution is the predictable output of that structural condition, representing not evidence of what these managers cannot do, but a reflection of what their environments do not permit them to do.

This review does not establish that interpretation as a causal claim. It proposes it as a theoretically grounded account of a pattern that the evidence base, taken as a whole, supports. Whether the mechanisms that produce this pattern operate as described, and how they vary across professional groups, healthcare systems, and cultural contexts, are empirical questions that primary research in defined settings is better positioned to address.

The practical implication is specific. Strengthening clinical leadership competency in the domains of greatest deficit requires investment at three levels simultaneously: individual preparation before and during role assumption, organisational reform that creates the enabling conditions for strategic competency expression, and system-level governance and workforce planning that treats leadership development as a structural priority rather than an individual responsibility.

Leadership competency should not be treated as an individual attribute. It is a contextually embedded capability whose development and expression depend on the interaction between what individuals can do and what their environments permit them to do.

## Figures and Tables

**Table 1:** Eligibility criteria mapped to the SPIDER framework.

SPIDER Items	Inclusion Criteria	Exclusion Criteria
Sample (S)	Clinical managers (nurses, physicians, allied health) holding formal leadership, supervisory, or administrative roles. Conditional Inclusion: Early-career doctors, residents, or public health managers only if evaluated within active institutional care settings (hospitals/clinics) regarding formal management duties.	Individuals without formal leadership or supervisory roles. General students/trainees without assigned management duties. Non-clinical hospital administrators. Macro-level public health policy or legislative leaders operating outside clinical delivery environments. Studies strictly focused on technical/clinical skills.
Phenomenon of interest (PI)	Leadership competency among clinical managers, including perceived leadership capability, contextual challenges affecting leadership practice, and leadership development needs associated with leadership roles.	Non-leadership competencies, including clinical competence, technical clinical skills, general service delivery only, or competency frameworks unrelated to leadership.
Design (D)	Primary empirical studies employing qualitative, quantitative, or mixed-methods design.	Secondary studies, including systematic or narrative reviews, commentaries, letters, dissertation, conference abstract, case report and grey literature.
Evaluation (E)	Empirical findings reporting leadership competency, perceptions, challenges, or leadership development needs.	Studies not reporting leadership-related outcomes or not providing extractable findings relevant to the review questions.
Research Type (R)	Qualitative, quantitative, and mixed-methods research	Conceptual papers, opinion pieces, protocols

**Table 2:** Summary of perceived leadership competency across CLCF domains.

CLCF Domain	Reported as Strength	Reported as Area of Weakness
Demonstrating Personal Qualities	Reported as strengths across multiple studies: integrity; professionalism; self-management; ethical and humane qualities	Reported as areas of variability in fewer studies: gaps in self-management, autonomy, and leadership maturity
Working with Others	Reported as strengths across multiple studies: communication; teamwork; relationship management; interpersonal collaboration	Reported as areas of limitation in fewer studies: conflict management; influencing others; complex interpersonal dynamics
Managing Services	Reported as strengths in fewer studies: basic operational management; planning; controlling; execution	Reported as areas of weakness across multiple studies: financial management; human resource management; resource allocation; performance management
Improving Services	Reported as strengths in fewer studies: quality improvement; performance improvement; clinical outcome management	Reported as areas of weakness across multiple studies: innovation; change management; service improvement; system transformation
Setting Direction	Reported as strengths in fewer studies: decision-making (operational level); problem-solving	Reported as areas of weakness across the greatest number of studies: strategic thinking; planning; vision setting; policy development; system-level leadership

**Footnote**: Classifications reflect qualitative synthesis of the extracted data (Supplementary Table S4) based on the number of contributing studies, not statistical frequency counts. Competencies were mapped to CLCF domains based on functional alignment. The relative prominence of interpersonal competencies may partly reflect measurement bias inherent in the instruments used across included studies.

**Table 3:** Summary of reported leadership challenges across included studies.

Challenge Category	Commonly Reported Challenges
Training and preparation challenges	Lack of formal leadership training Inadequate preparation before assuming leadership roles Limited access to structured leadership development programmes
Organisational and structured challenges	Bureaucracy Limited decision-making authority Weak reward systems Unclear organisational structures Governance constraints
Role-related challenges	Role ambiguity Tension between strategic and operational responsibilities Dual clinical-managerial expectations Managing peers Unclear professional boundaries
Resources and workload challenges	Heavy workload Staffing shortages Time constraints Limited institutional support
Relational and professional challenges	Conflict management difficulties Lack of support from colleagues or supervisors Professional tensions Resistance to change Managing difficult interpersonal situations
Socio-cultural and contextual challenges	Hierarchical culture Political pressure Gender bias Patriarchy Informal power networks Cultural expectations shaping leadership behaviour

**Footnote**: Challenge categories were developed through qualitative synthesis of the challenges reported across the included studies presented in Supplementary Table S3.

**Table 4:** Summary of reported leadership development needs across included studies.

Development Area	Commonly Reported Needs
Formal leadership training and structured development	Formal leadership training Competency-based leadership development programmes Structured preparation before or during transition to leadership roles
Strategic and managerial development	Financial management Human resources management Information management Policy and system knowledge Strategic planning Governance Business literacy Resources and services management
Interpersonal and communication development	Communication skills Teamwork Conflict management Feedback skills Relationship building Influencing and negotiation
Mentoring, coaching, and support mechanisms	Mentoring Coaching Peer learning Leadership networks Supportive team culture Experiential learning
Continuous professional development and reflective learning	Ongoing training Continue professional development Lifelong learning Leadership pathways Succession planning
Organisational and system-level support	Organisational support Enabling leadership environments Governance reform Protected time Flexible policy Equality and inclusion initiatives

**Footnote**: Development areas reported across the included studies fall into three distinct categories: (1) competency domains requiring development (e.g., strategic thinking, financial management); (2) delivery mechanisms through which development is facilitated (e.g., mentoring, coaching, peer learning); and (3) enabling conditions that must be present for development to be effective (e.g., organisational support, protected time). These categories are not mutually exclusive. Data were derived from the extracted evidence presented in Supplementary Table S3.

## Data Availability

No new data were created or analysed in this study.

## Supplementary Materials

The following supporting information can be downloaded at: https://www.mdpi.com/article/10.3390/healthcare14121720/s1, Table S1. Search strategy for four databases. Table S2. Quality assessment for qualitative studies. Table S3. Characteristics of included studies. Table S4. Mapping of reported leadership competencies from included studies to the domains of the CLCF.

## References

[1] WHO Everybody’s Business—Strengthening Health Systems to Improve Health Outcomes. https://www.who.int/publications/i/item/everybody-s-business----strengthening-health-systems-to-improve-health-outcomes.

[2] OECD (2019). Health in the 21st Century: Putting Data to Work for Stronger Health Systems.

[3] West M., Armit K., Loewenthal L., Eckert R., West T., Lee A. (2015). Leadership and Leadership Development in Health Care: The Evidence Base.

[4] Institute of Medicine (US) Committee on Quality of Health Care in America (2001). Crossing the Quality Chasm: A New Health System for the 21st Century.

[5] World Health Organization (2019). Patient Safety: Global Action on Patient Safety.

[6] Slawomirski L., Auraaen A., Klazinga N. (2017). The Economics of Patient Safety.

[7] Currie G., Lockett A. (2011). Distributing Leadership in Health and Social Care: Concertive, Conjoint or Collective?. Int. J. Manag. Rev..

[8] Denis J.-L., Langley A., Sergi V. (2012). Leadership in the Plural. Annals.

[9] Hartley J., Benington J. (2010). Introducing Leadership. Leadership for Healthcare.

[10] Kyriakidou N., Aspasia G., George P., Anastasios S., Marios A. (2021). Leadership Development in Health Care: The Role of Clinical Leaders. J. Hum. Resour. Sustain. Stud..

[11] Le Deist F.D., Winterton J. (2005). What Is Competence?. Hum. Resour. Dev. Int..

[12] Boyatzis R.E. (2008). Competencies in the 21st Century. J. Manag. Dev..

[13] NHS. Institute for Innovation and Improvement and Academy of Medical Royal Colleges (2010). Medical Leadership Competency Framework: Enhancing Engagement in Medical Leadership.

[14] NHS Leadership Academy (2011). Clinical Leadership Competency Framework.

[15] American Organization for Nursing Leadership (2022). AONL Nurse Leader Core Competencies.

[16] National Center for Healthcare Leadership (2018). Health Leadership Competency Model 3.0.

[17] Howard P.F., Liang Z., Leggat S., Karimi L. (2018). Validation of a Management Competency Assessment Tool for Health Service Managers. J. Health Organ. Manag..

[18] Greenhalgh T., Robert G., Macfarlane F., Bate P., Kyriakidou O. (2004). Diffusion of Innovations in Service Organizations: Systematic Review and Recommendations. Milbank Q..

[19] Best A., Greenhalgh T., Lewis S., Saul J.E., Carroll S., Bitz J. (2012). Large-System Transformation in Health Care: A Realist Review. Milbank Q..

[20] Mumford M.D., Zaccaro S.J., Harding F.D., Jacobs T.O., Fleishman E.A. (2000). Leadership Skills for a Changing World: Solving Complex Social Problems. Leadersh. Q..

[21] Biddle B.J. (2013). Role Theory: Expectations, Identities, and Behaviors.

[22] Peters L.H., O’Connor E.J. (1980). Situational Constraints and Work Outcomes: The Influences of a Frequently Overlooked Construct. Acad. Manag. Rev..

[23] Page M.J., McKenzie J.E., Bossuyt P.M., Boutron I., Hoffmann T.C., Mulrow C.D., Shamseer L., Tetzlaff J.M., Akl E.A., Brennan S.E. (2021). The PRISMA 2020 Statement: An Updated Guideline for Reporting Systematic Reviews. Int. J. Surg..

[24] Cooke A., Smith D., Booth A. (2012). Beyond PICO: The SPIDER Tool for Qualitative Evidence Synthesis. Qual. Health Res..

[25] Hong Q.N., Fàbregues S., Bartlett G., Boardman F., Cargo M., Dagenais P., Gagnon M.-P., Griffiths F., Nicolau B., O’Cathain A. (2018). The Mixed Methods Appraisal Tool (MMAT) Version 2018 for Information Professionals and Researchers. Educ. Inf..

[26] Giri P., Aylott J., Kilner K. (2017). Self-Determining Medical Leadership Needs of Occupational Health Physicians. Leadersh. Health Serv..

[27] McGowan E., Walsh C., Stokes E. (2017). Physiotherapy Managers’ Perceptions of Their Leadership Effectiveness: A Multi-Frame Analysis. Physiotherapy.

[28] Welch T., Glenn C. (2022). Nursing Leadership in Rural Hospitals: A Competency Needs Assessment. Online J. Rural. Nurs. Health Care.

[29] Mrayyan M.T. (2022). Correlates and Predictors of Clinical Leadership Need Analysis (CLeeNA) for Nurses: A Cross-Sectional Web-Based Study. Nurs. Forum.

[30] Pokhrel P., Jones A., Crowe M., Kaphle H., Liang Z. (2025). Assessment of Management Competency Among Senior Hospital Managers in Nepalese Public Hospitals: A Cross-Sectional Study. Asia Pac. J. Health Manag..

[31] Gulati K., Singh A., Gupta S., Sarkar C. (2022). Strengthening Leadership Capacity: An Unaddressed Issue in Indian Healthcare System. Leadersh. HEALTH Serv..

[32] Isibor E., Kanmodi K., Adebayo O., Olaopa O., Igbokwe M., Adufe I., Oduyemi I., Adeniyi M., Oiwoh S., Omololu A. (2020). Exploring Issues and Challenges of Leadership among Early Career Doctors in Nigeria Using a Mixed-Method Approach: CHARTING Study. Eur. J. Investig. HEALTH Psychol. Educ..

[33] Liou Y.-F., Liaw J.-J., Chang Y.-C., Kao J.-H., Feng R.-C. (2021). Psychometric Properties and Development of the Competency Inventory for Taiwanese Nurse Managers across All Levels. J. Nurs. Manag..

[34] Dixon-Woods M., Agarwal S., Jones D., Young B., Sutton A. (2005). Synthesising Qualitative and Quantitative Evidence: A Review of Possible Methods. J. Health Serv. Res. Policy.

[35] Whittemore R., Knafl K. (2005). The Integrative Review: Updated Methodology. J. Adv. Nurs..

[36] Backman A., Sundström M., Jeon Y., Edberg A. (2025). Framing Person-Centred Leadership in Residential Care: A Cross-Cultural Adaptation of the Aged-Care Clinical Leadership Qualities Framework. J. Clin. Nurs..

[37] Bigbee J.L., Otterness N., Gehrke P. (2010). Public Health Nursing Competency in a Rural/Frontier State. Public. Health Nurs..

[38] Fanelli S., Pratici L., Zangrandi A. (2022). Managing Healthcare Services: Are Professionals Ready to Play the Role of Manager?. Health Serv. Manag. Res..

[39] Fraser T.N., Blumenthal D.M., Bernard K., Iyasere C. (2015). Assessment of Leadership Training Needs of Internal Medicine Residents at the Massachusetts General Hospital. Bayl. Univ. Med. Cent. Proc..

[40] Gulati K., Madhukar V., Verma V., Singh A.R., Gupta S.K., Sarkar C. (2019). Medical Leadership Competencies: A Comparative Study of Physicians in Public and Private Sector Hospitals in India. Int. J. Health Plan. Manag..

[41] Gulati K., Sarkar C., Verma V., Singh A.R., Gupta S.K. (2021). Assessment of Medical Leadership Competencies and Development Needs: First Comprehensive Study from India. Int. J. Healthc. Manag..

[42] Liang Z., Howard P., Wang J., Xu M. (2020). A Call for Leadership and Management Competency Development for Directors of Medical Services-Evidence from the Chinese Public Hospital System. Int. J. Environ. Res. Public. Health.

[43] Liang Z., Howard P., Wang J., Xu M., Zhao M. (2020). Developing Senior Hospital Managers: Does “One Size Fit All”?—Evidence from the Evolving Chinese Health System. BMC Health Serv. Res..

[44] Moreno R. (2025). Factors Associated with Healthcare Leaders’ Perceived Self-Efficacy During Crises. J. Healthc. Manag..

[45] Patnaik S.K., Sahran D., Jithesh V., Garg N., Misra A., Mishra S., Kumar V., Shukla S. (2026). Competency Assessment of Healthcare Leaders. J. Mar. Med. Soc..

[46] Pillay R. (2011). The Skills Gap in Nursing Management in the South African Public Health Sector. Public. Health Nurs..

[47] Tung J., Nahid M., Rajan M., Bogdewic S., Mancuso C. (2025). Enhancing a Faculty Development Program: Identifying and Addressing Leadership Skill Gaps Using an Established Leadership Framework. J. Healthc. Leadersh..

[48] Warshawsky N., Cramer E. (2019). Describing Nurse Manager Role Preparation and Competency: Findings from a National Study. J. Nurs. Adm..

[49] Iblasi A.S., Makahleh S., Aungsuroch Y., Gunawan J., Juanamasta I.G. (2024). First-Line Nurse Managerial Competence and Its Influencing Factors in Public Jordanian Hospitals. Nurse Media J. Nurs..

[50] Ibrahim R., Charar J., Majed M., Ayaad O. (2025). Physician Leadership Competency: A Quantitative Study of Challenges, Barriers and Development Models. Br. J. Healthc. Manag..

[51] Jalghef M., Eshah N., Al-Oweidat I., Nashwan A. (2023). Self-Perceived Performance-Based Training Needs among Middle-Level Nursing Managers in Jordan. Int. J. Healthc. Manag..

[52] Miltner R.S., Jukkala A., Dawson M.A., Patrician P.A. (2015). Professional Development Needs of Nurse Managers. J. Contin. Educ. Nurs..

[53] Furunes T., Kaltveit A., Akerjordet K. (2018). Health-Promoting Leadership: A Qualitative Study from Experienced Nurses’ Perspective. J. Clin. Nurs..

[54] Hartviksen T., Aspfors J., Uhrenfeldt L. (2020). Healthcare Middle Managers’ Capacity and Capability to Quality Improvement. Leadersh. Health Serv..

[55] Dopelt K., Levi B., Davidovitch N. (2021). Identifying Distinctive Traits of Healthcare Leaders in Israel: In-Depth Interviews with Senior Physicians—An Exploratory Study. Leadersh. Health Serv..

[56] Mai D.H., Newton H., Farrell P.R., Mullan P., Kapoor R. (2021). Assessment of Clinical Leadership Training Needs in Senior Pediatric Residents. J. Med. Educ. Curric. Dev..

[57] Xu X., Zhang Y., Zhou P., Zhou X. (2022). Developing a Leadership and Management Competency Framework for Nurse Champion: A Qualitative Study from Shanghai, China. J. Nurs. Manag..

[58] Ylitalo A., Laukka E., Heponiemi T., Kanste O.I. (2022). Primary Healthcare Managers’ Perceptions of Management Competencies at Different Management Levels in Digital Health Services: Secondary Analysis. Leadersh. Health Serv..

[59] Kelly D., Horseman Z., Strachan F., Hamilton S., Jones A., Holloway A., Rafferty A., Noble H., Reid J., Harris R. (2023). Strengthening the Role of the Executive Nurse Director: A Qualitative Interview Study. J. Adv. Nurs..

[60] Gulati K., Davies J., Gonzalez de la Fuente A., Singh A.R. (2024). Striving for Equity: Exploring Gender-Inclusive Medical Leadership in India. BMJ Lead..

[61] Gaudet R., Lord M.-M., Maclure J., Drolet M.-J. (2025). Why Become a Healthcare Manager? Ethically Reflecting on the Path to Leadership of Public and Private Sectors’ Occupational Therapists of Quebec-Canada. Occup. Ther. Health Care.

[62] Hodza-Beganovic R., Berggren P., Edelbring S. (2025). The Role of Leadership in Enhancing Non-Technical Skills in Healthcare: A Qualitative Study in a Balkan Context. Hum. Resour. Health.

[63] Hussein H.S., Bahmanpour K., Fathi M., Fatemi A. (2025). Understanding Critical Thinking Practices in Iranian Healthcare Managers: Qualitative Insights. Health Promot. Perspect..

[64] Kämäräinen P., Mikkola L., Nurmeksela A., Kvist T. (2025). Nurse Leaders’ Perceptions of Development of Their Own Interpersonal Communication Competence: A Qualitative Descriptive Study in Social and Healthcare Organisations. J. Adv. Nurs..

[65] Matandela M., Chisale G.L., Matahela V.E. (2025). Nurse Leaders’ Perceptions of Leadership Development Needs for Strengthening the Nursing Workforce: A South African Pilot Study. Front. Health Serv..

[66] Al Ansari A.M. (2026). Managerial Perceptions of Core Competencies for Healthcare Middle Managers in Bahrain and Saudi Arabia: A Qualitative Study. Int. J. Med. Educ..

[67] Ireri S., Walshe K., Benson L., Mwanthi M.A. (2011). A Qualitative and Quantitative Study of Medical Leadership and Management: Experiences, Competencies, and Development Needs of Doctor Managers in the United Kingdom. Int. J. Healthc. Manag..

[68] Ireri S.K., Walshe K., Benson L., Mwanthi M. (2017). A Comparison of Experiences, Competencies and Development Needs of Doctor Managers in Kenya and the United Kingdom (UK). Int. J. Health Plan. Manag..

[69] Dawson M., Phillips B., Leggat S.G. (2013). Effective Clinical Supervision for Regional Allied Health Professionals: The Supervisor’s Perspective. Aust. Health Rev..

[70] Dickinson H., Ham C., Snelling I., Spurgeon P. (2013). Medical Leadership Arrangements in English Healthcare Organisations: Findings from a National Survey and Case Studies of NHS Trusts. Health Serv. Manag. Res..

[71] Liang Z., Leggat S.G., Howard P.F., Koh L. (2013). What Makes a Hospital Manager Competent at the Middle and Senior Levels?. Aust. Health Rev..

[72] Fiset V., Luciani T., Hurtubise A., Grant T.L. (2017). Clinical Nursing Leadership Education in Long-Term Care. J. Gerontol. Nurs..

[73] Shikama Y., Oikawa S., Stanyon M., Yasuda M., Otani K. (2024). Culturally-Aligned Clinical Leadership Competencies for Effective Teamwork in Japanese Healthcare. BMC Med. Educ..

[74] Gunter S., Nogueira R., Hudson C., Morton R., Jones C. (2025). Perceptions of Sustainable Leadership in Australian Healthcare. J. Healthc. Leadersh..

[75] Rosser E.A., Russell K., Ng Y.C., Lott T.F., Buckner E. (2025). Sustaining Success: The Evolving Impact of a Global Leadership Mentoring Programme. Int. Nurs. Rev..

[76] Van Der Berg-Cloete S., Tosh C.A., Buch E. (2025). External Factors Affecting the Efficacy of the Albertina Sisulu Executive Leadership Programme in Health Fellowship in South Africa: A 360° Qualitative Assessment. Afr. J. Health Prof. Educ..

[77] Gulati K., Singh A., Kumar S., Verma V., Gupta S., Sarkar C. (2019). Impact of a Leadership Development Programme for Physicians in India. Leadersh. Health Serv..

