# Supplementary material for: Identifying Clinical Managers’ Leadership Competencies: A Systematic Review and Cross-Frameworks Mapping Using the CLCF
Source: Healthcare (Basel). 2026 Jun 15;14(12):1720. doi: 10.3390/healthcare14121720 (PMC13299240; doi:10.3390/healthcare14121720)
Supplement: Supplementary file 1 [file healthcare-14-01720-s001.zip › healthcare-4275318-supplementary.pdf]

Supplementary Table S1. Search strategy for four databases

Databases	Search strategy	Records
**Ovid MEDLINE**	Ovid MEDLINE(R) ALL <1946 to February 12, 2026><br><br>1 ((Healthcare or Hospital or Medical or Nurse* or "Clinic* or Health professional*" or physician* or doctor* or pharmacist* or physiotherapist*) adj2 (Director* or Leader* or Executive* or Supervisor* or Chief* or Manager*)).mp. [mp=title, book title, abstract, original title, name of substance word, subject heading word, floating sub-heading word, keyword heading word, organism supplementary concept word, protocol supplementary concept word, rare disease supplementary concept word, unique identifier, synonyms, population supplementary concept word, anatomy supplementary concept word] 39189<br>2 head of department.mp. [mp=title, book title, abstract, original title, name of substance word, subject heading word, floating sub-heading word, keyword heading word, organism supplementary concept word, protocol supplementary concept word, rare disease supplementary concept word, unique identifier, synonyms, population supplementary concept word, anatomy supplementary concept word] 145<br>3 ((Skill or learning or training or education or Competenc* or Proficiency or Developmental) adj2 (requirement* or gap* or need* or development* or priorities)).mp. 510009<br>4 (Professional growth or Capacity building need*).mp. [mp=title, book title, abstract, original title, name of substance word, subject heading word, floating sub-heading word, keyword heading word, organism supplementary concept word, protocol supplementary concept word, rare disease supplementary concept word, unique identifier, synonyms, population supplementary concept word, anatomy supplementary concept word] 2010<br>5 (leadership or management).mp. [mp=title, book title, abstract, original title, name of substance word, subject heading word, floating sub-heading word, keyword heading word, organism supplementary concept word, protocol supplementary concept word, rare disease supplementary concept word, unique identifier, synonyms, population supplementary concept word, anatomy supplementary concept word] 2058234<br>6 1 or 2 39324<br>7 3 or 4 511866<br>8 5 and 6 and 7 711<br>9 limit 8 to yr="2010 -Current" 537	537
**EMCARE**	Emcare 1995 to present<br><br>1 ((Healthcare or Hospital or Medical or Nurs* or "Clinic* or Health professional*" or physician* or doctor* or pharmacist* or	719

	physiotherapist*) adj2 (Director* or Leader* or Executive* or Supervisor* or Chief* or Manager*)).mp. [mp=title, abstract, heading word, drug trade name, original title, device manufacturer, drug manufacturer, floating subheading word, device trade name, keyword heading word, candidate term word] 31851<br>2 head of department.mp. [mp=title, abstract, heading word, drug trade name, original title, device manufacturer, drug manufacturer, floating subheading word, device trade name, keyword heading word, candidate term word] 72<br>3 ((Skill or learning or training or education or Competenc* or Proficiency or Developmental) adj2 (requirement* or gap* or need* or development* or priorities)).mp. 148647<br>4 (Professional growth or Capacity building need*).mp. [mp=title, abstract, heading word, drug trade name, original title, device manufacturer, drug manufacturer, floating subheading word, device trade name, keyword heading word, candidate term word] 1597<br>5 (leadership or management).mp. [mp=title, abstract, heading word, drug trade name, original title, device manufacturer, drug manufacturer, floating subheading word, device trade name, keyword heading word, candidate term word] 1061880<br>6 1 or 2 31917<br>7 3 or 4 150123<br>8 5 and 6 and 7 866<br>9 limit 8 to yr="2010 - 2026" 719	
**WOS**	2: (TS=(((Healthcare OR Hospital OR Medical OR Nurs* OR "Clinic* or Health professional*" OR physician* OR doctor* OR pharmacist* OR physiotherapist*) NEAR/2 (Director* OR Leader* OR Executive* OR Supervisor* OR Chief* OR Manager*)))) OR TS=("head of department")<br><br>3: TS=((Skill OR learning OR training OR education OR Competenc* OR Proficiency OR Developmental) NEAR/2 (requirement* OR gap* OR need* OR development* OR priorities)) OR TS=("Professional growth" or "Capacity building need*")<br>4: TS=(leadership or management)<br>5: #4 AND #3 AND #2 Results: 1530	1530
**CINAHL**	((Healthcare OR Hospital OR Medical OR Nurs* OR Clinic* OR Health professional* OR physician* OR doctor* OR pharmacist*) N2 (Director* OR Leader* OR Executive* OR Supervisor* OR Chief* OR Manager*)) AND ((Skill OR learning OR training OR education OR Competenc* OR Proficiency OR Developmental) N2 (requirement* OR gap* OR need* OR development* OR priorities)) AND (leadership OR management)	1137

Supplementary Table S2. Quality assessment for qualitative studies

Studies	1 - Are there clear research questions?	2 - Do the collected data allow to address the research questions?	3- Is the qualitative approach appropriate to answer the research question?	4 - Are the qualitative data collection methods adequate to address the research question?	5 - Are the findings adequately derived from the data?	6 - Is the interpretation of results sufficiently substantiated by data?	7 - Is there coherence between qualitative data sources, collection, analysis and interpretation?
(Al Ansari, 2026)	Yes	Yes	Yes	Yes	Yes	Yes	Yes
(Dopelt, Levi and Davidovitch, 2021)	Yes	Yes	Yes	Yes	Yes	Yes	Yes
(Furunes, Kaltveit and Akerjordet, 2018)	Yes	Yes	Yes	Yes	Yes	Yes	Yes
(Gaudet *et al.*, 2025)	Yes	Yes	Yes	Yes	Yes	Yes	Yes
(Gulati *et al.*, 2024)	Yes	Yes	Yes	Yes	Yes	Yes	Yes
(Hartviksen, Aspfors and Uhrenfeldt, 2020)	Yes	Yes	Yes	Yes	Yes	Yes	Yes
(Hodza-Beganovic, Berggren and Edelbring, 2025)	Yes	Yes	Yes	Yes	Yes	Yes	Yes

(Hussein *et al.*, 2025)	Yes	Yes	Yes	Yes	Yes	Yes	Yes
(Kämäräinen *et al.*, 2025)	Yes	Yes	Yes	Yes	Yes	Yes	Yes
(Kelly *et al.*, 2023)	Yes	Yes	Yes	Yes	Yes	Yes	Yes
(Mai *et al.*, 2021)	Yes	Yes	Yes	Yes	Yes	Yes	Yes
(Matandela, Chisale and Matahela, 2025)	Yes	Yes	Yes	Yes	Yes	Yes	Yes
(Miltner *et al.*, 2015)	Yes	Yes	Yes	Yes	Yes	Yes	Yes
(Xu *et al.*, 2022)	Yes	Yes	Yes	Yes	Yes	Yes	Yes
(Ylitalo *et al.*, 2022)	Yes	Yes	Yes	Yes	Yes	Yes	Yes
Studies	1 - Are there clear research questions?	2 - Do the collected data allow to address the research questions?	3 - Is the sampling strategy relevant to address the research question?	4 - Is the sample representative of the target population?	5 - Are the measurements appropriate?	6 - Is the risk of nonresponse bias low?	7 - Is the statistical analysis appropriate to answer the research question?
(Backman *et al.*, 2025)	Yes	Yes	Yes	CT	Yes	Yes	Yes
(Bigbee, Otterness and Gehrke, 2010)	Yes	Yes	Yes	Yes	Yes	CT	Yes

(Fanelli, Pratici and Zangrandi, 2022)	Yes	Yes	Yes	CT	Yes	CT	Yes
(Fraser *et al.*, 2015)	Yes	Yes	Yes	CT	Yes	CT	Yes
(Giri, Aylott and Kilner, 2017)	Yes	Yes	Yes	CT	Yes	No	Yes
(Gulati *et al.*, 2019)	Yes	Yes	Yes	Yes	Yes	CT	Yes
(Gulati *et al.*, 2021)	Yes	Yes	Yes	Yes	Yes	CT	Yes
(S Iblasi *et al.*, 2024)	Yes	Yes	Yes	CT	Yes	CT	Yes
(Ibrahim *et al.*, 2025)	Yes	Yes	CT	CT	Yes	Yes	Yes
(Jalghef *et al.*, 2023)	Yes	Yes	Yes	CT	Yes	Yes	Yes
(Liang, Howard, Wang and Xu, 2020a)	Yes	Yes	Yes	Yes	Yes	Yes	Yes
(Liang, Howard, Wang, Xu, *et al.*, 2020b)	Yes	Yes	Yes	Yes	Yes	Yes	Yes

(McGowan, Walsh and Stokes, 2017)	Yes	Yes	Yes	CT	Yes	No	Yes
(Moreno, 2025)	Yes	Yes	Yes	CT	Yes	CT	Yes
(Mrayyan, 2022)	Yes	Yes	CT	No	Yes	CT	Yes
(Patnaik *et al.*, 2026)	Yes	Yes	Yes	CT	Yes	Yes	Yes
(Pillay, 2011)	Yes	Yes	Yes	CT	Yes	Yes	Yes
(Pokhrel *et al.*, 2025)	Yes	Yes	CT	No	Yes	CT	Yes
(Tung *et al.*, 2025)	Yes	Yes	Yes	Yes	Yes	CT	Yes
(Warshawsky and Cramer, 2019)	Yes	Yes	Yes	Yes	Yes	CT	Yes
(Welch, 2022)	Yes	Yes	Yes	CT	Yes	No	Yes
Studies	1 - Are there clear research questions?	2 - Do the collected data allow to address the research questions?	3 - Is there an adequate rationale for using a mixed methods design to address the research question?	4 - Are the different components of the study effectively integrated to answer the research question?	5 - Are the outputs of the integration of qualitative and quantitative components adequately interpreted?	6 - Are divergences and inconsistencies between quantitative and qualitative results adequately addressed?	7 - Do the different components of the study adhere to the quality criteria of each tradition of the methods involved?

(Dawson *et al.*, 2013)	Yes	Yes	Yes	Yes	Yes	CT	Yes
(Dickinson *et al.*, 2013)	Yes	Yes	Yes	Yes	Yes	Yes	Yes
(Fiset *et al.*, 2017)	Yes	Yes	Yes	Yes	Yes	CT	Yes
(Gulati *et al.*, 2022)	Yes	Yes	CT	No	No	No	No
(Gunter *et al.*, 2025)	Yes	Yes	Yes	CT	CT	CT	Yes
(Ireri *et al.*, 2011)	Yes	Yes	Yes	Yes	Yes	Yes	Yes
(Ireri *et al.*, 2011)	Yes	Yes	Yes	Yes	Yes	Yes	Yes
(Isibor *et al.*, 2020)	Yes	Yes	Yes	No	No	No	Yes
(Liang *et al.*, 2013)	Yes	Yes	Yes	Yes	Yes	Yes	Yes
(Liou *et al.*, 2021)	Yes	Yes	Yes	Yes	Yes	No	Yes
(Rosser *et al.*, 2025)	Yes	Yes	Yes	Yes	Yes	Yes	Yes
(Shikama *et al.*, 2024)	Yes	Yes	Yes	Yes	Yes	CT	Yes

(Van Der Berg-Cloete, Tosh and Buch, 2025)	Yes	Yes	Yes	Yes	Yes	CT	CT

Supplementary Table S3. Characteristics of Included Studies .

First Author	Year	Country	Journal	Settings	Population	Sample	Age	Design	Scale	Year of Experience	Framework	High Competencies & Perception	Low Competencies & Perception	Challenges	Developmental Needs
Liang	2020	China	International Journal of Research and Environmental Public Health	Chinese Public Hospital System	Directors & Deputy directors of Medical Service	295	47.2	Three-component survey: cross-sectional & descriptive	Self-rating	1 years	Management competency assessment program (MCAP): Six core competencies: Evidence, Resources, Knowledge, communication, leadership and change	NR	Low self-perceived competency; conflict management; performance management and improvement; new skills development; making decision and making change	Inadequate formal and informal management training prior to leadership role	The study suggests two system level approaches (health and higher education systems) and one organization level approach

Ireri	2011	Kenya	International Journal Of Health Planning And Management	Kenya and UK healthcare	Doctors managers	202	NR	Mixed-method	Self-rating	1 years	Medical Leadership Competency Framework (Personal qualities, working with others, managing service, improving service, and setting direction)	High self perceived competencies in personal qualities, working with others and managing service	Low self perceived competencies in service improvement and working with others, self-management by Kenya doctors,	Lack of leadership training; weak reward system (kenya)	Training and developmental needs in managing service (financial management, HRM, information management) and working with others (motivation & conflict management)
Ireri	2017	Kenya	Journal of Management & Marketing in Healthcare	United Kingdom NHS	Doctors managers	327	30-64	Mixed-method	Self-rating	2 years	Medical Leadership Competency Framework (Personal qualities, working with others, managing service, improving service, and setting direction)	Managing services, working with others, and personal qualities	Improving services and setting direction	People management issues, time management, managing peers, governance, financial, assumptions, isolation, and cost of being in that position; inadequate prior preparation for management role; persuasion to join managemen	Clinical credibility; Financial management skills were the most needed skills; human resource management, which included leadership skills, persuasion techniques, mentoring, conflict management, and coaching

														t; work overload; limited resources, Lack of power/authority, unclear boundaries, conflicts between personal and organizational values, conflicts between doctors and managers	
Mai	2021	USA	Journal of Medical Education and Curricular Development	US healthcare	Senior pediatric residents	66	NR	Qualitative	Self-rating	3 years	NR	NA	NA	Lack of Leadership training	(1) effective and timely communication, (2) teaching strategies in the clinical setting, (3) effective time management and resource utilization, and (4) self-care and emotional regulation techniques to build resilience.
Fraser	2015	USA	Baylor University Medical	Massachusetts Gene	Internal medicine residents	55	NR	Quantitative	Self-rating	1 years	NR	NA	NA	Lack formal leadership training	Formal leadership training: “leading a team” (98% of residents),

			Center Proceedings	ral Hos											"innovative problem solving" (93%), "coaching and developing others" (93%), and "resolving interpersonal conflict" (84%), time management, self management, Embracing change, Self-awareness
Gulati	2021a	India	International Journal of Healthcare Management	Private and Public Hospital in India	Doctors managers	364	25-78	Quantitative	Self-assessed proficiency	14.5 Years	Medical Leadership Competency Framework (Personal qualities, working with others, managing service, improving service, and setting direction)	NR	Setting directions, Working with others, and Managing services, with the lowest rated competency being 'Knowledge of HR, procurement,financial and contracts management'	Formal management training, Speciality and Duration of management/leadership experience	Physicians indicated need for medical leadership development program at all level
Fiset	2017	Canada	Journal of Gerontological nursing	Long-term care (LTC)	RNs, registered practical nurses,	86	NR	Mixed methods	Self-assessed proficiency	NR	NR	NA	NA	Lack of Leadership training	Interpersonal: communication, inspiration/motivation, conflict resolution,

				homes	and nursing administrators										relationship building, and self awareness; Organization: strategic planning, policy and program development, team building, and change theory; Management: Recruitment and retention, human resources policies and procedures, regulatory compliance, financial and budgetary planning, supervision and mentoring, and quality improvement
Liang	2013	Australia	Australian Journal of Primary Health	Australia community health services	Medical managers	90	NR	mixed-method	Self-assessed proficiency	NR	NR	NA	NA	Lack of Leadership training	Knowledge of health care environment and the organization; and Interpersonal, communication qualities and relationship management; ;Leading and managing change; and Evidence informed decision-making.; communication qualities; Self-

															management and resilience; Operations, administration and resource management
Mrayyan	2022	Jordan	Bursing Forum	Jordanian private and public hospital	Nurse managers	349	>35	Quantitative cross-sectional design	Self-assessed proficiency	>4 years	NR	Leadership and clinical practice	financial and service management	Lack of Leadership training	Self and team development; Staff and care delivery; Technology and care initiatives; Technology and care initiatives; Financial and service management; Leadership and clinical practice; Patient safety and risk management; Standards of care
Warshawsky	2019	USA	Journal of Nursing Administration	National Hospitals participating in National database of Nursing Quality	Nurse managers	647	45.33	Quantitative	Self-rating	>2 years	Nursing Manager Leadership Domain Framework: Science: Managing the business and Art: leading the people and leader within, and creating leader in your self	High competency perception at the first 6 years of managing role. And it includes: human resources management, clinical practices, diversity management, and technology	Financial management, strategic management, performance improvement, and foundational thinking	Lack of Leadership training and lack interest in management role	Need to develop strategies and policies that facilitates nurse managers competencies

				Indicators											
Liang	2020b	China	BMC Health Services Research	Hospitals in Jinan, Shandong Province, China	Nursing Directors, Clinical directors, Administrators,	513	45	Quantitative	self perceived competency	9.3	MCAP: Six core competencies: Evidence, Resources, Knowledge, communication, leadership and change	Evidence, Knowledge, communication, and leadership	Resources and Change from the 6 competency list. Conflict management, innovative team work, performance,	Inadequate formal and informal management training before commencing their management positions . However, clinical manager are more prepared than nurse managers	The study suggests that management training and support should be provided using a systematic approach with specific consideration to hospital types and management levels and positions.
Fanelli	2022	Italy	Health Services Management Research	Italian health organizations of Emilia-Romagna and Lombardy regions	Doctor, nurse or other health professionals	366	NR	Quantitative	self perceived competency	NR	NR	Problem solving, planning, operation and project management, leadership and internal communication, and quality improvement	Financial management, Human resource management, organizational design, External communication, output and outcome assessment	NR	Leadership competency need in the area of organizational design, HR management, quality improvement, leadership and internal communication

Dickinson	2013	UK	Health Services Management Research	NHS trusts	Medical leaders, managers and nurse leaders	151	NR	mixed method	Self-rated	NR	NR	NR	NR	Lack leadership training and uninterested in leadership role	Developing career structures to make it easier for doctors to take on leadership roles; providing training, development and support in management and leadership at different stages of doctors' careers; and ensuring that pay and other rewards are commensurate with the responsibilities of medical leaders
Gulati	2019	India	International Journal Of Health Planning And Management	Private and Public Hospital in India	Doctors leaders	532	38.8	Quantitative	Self-assessed proficiency	3.3 years	Medical Leadership Competency Framework (Personal qualities, working with others, managing service, improving service, and setting direction)	Personal Qualities and Improving service	Working with others, managing service, and setting direction. Items under this three checklist includes; knowledge of HR, procurement, finance, contract management, ability	Lack of Leadership training	Medical development program needs was identified by the study.

													to influence decision, time and stress management, conducting need analysis, identify and prioritize requirement.		
Welch	2022	USA	Online Journal of Rural Nursing and Health Care	Rural hospital in Alabama Black Belt Region	Nurse manager or nursing directors	22	NR	Qualitative	Self-assessed proficiency	>5 years	AONE: Nurse Manager Competencies Self-Assessment Inventory Tool: (a) managing the business, (b) leading the people, and (c) creating the leader within yourself	Nurse with >5 years of leader experience were more competent	Nurse leaders with <5 years of leadership experience are less proficient in knowledge of quality improvement strategies, ability to lead multi-generational work teams (communication style, motivation	Years of experience	Identified need for leadership development and commitment of nurse leaders in leadership role to be able to develop the necessary leadership continence

													factors, conflict management, or even work expectations)		
Miltner	2015	USA	Journal of Continue Education In Nursing	US healthcare	Nurse managers	20	45.1	Qualitative	Self-assessed proficiency	20.1	AONE: Nurse Manager Competencies Self-Assessment Inventory Tool: (a) managing the business, (b) leading the people, and (c) creating the leader within yourself	NR	Managing Versus Leading, Gaining a Voice, and Garnering Support	Understanding organizational behavior, use of data to make decisions, and refined problem-solving skills	Professional development activities focusing on higher level leadership competencies could assist managers to be more successful in this challenging, but critical, role
Liou	2021	Taiwan	Journal of Nursing Management	Taiwan Healthcare settings	Nurse managers	573	41-50	Mixed methods	Self-assessed proficiency	11.6 years	NR	NA	NA	Lack leadership competency skills	The study identify four leadership competency needs with several items. 1). Leadership (advocacy, strategic thinking, establishing policy, system and structure, being politically astute, external awareness, leading change, leading on vision and values, global perspective and mindset); 2).

															Business literacy (performance improvement, financial management, working knowledge of information management and technology, evidence based decision making skills, creativity and innovation, and setting direction); 3). Integrative skills (Planning and organization, quality and safety orientation, execution, problem solving, and analytic thinking) 4). Communication and relationship (effective communication, relationship building and management, interpersonal skills, influencing and negotiation)
Bigbee	2010	USA	Public Health Nursing	Boise healt hcare	Nurse manager s	124	49.2	Quantitat ive		6 years	8 domains of public health competency: analytic	communication, cultural	Policy developme nt/program planning,	Lack leadership	The paper identified need for policy development/progr

				settings							assessment skills, policy development/program planning skills, communication skills, cultural competency skills, community dimensions of practice skills, basic public health sciences skills, leadership and systems thinking skills, and fifinancial planning and management skills.	competency, and leadership skills	analytic assessment, and fifinancial planning/ management skills	competency skills	am planning skills, analytic assessment skills, and fifinancial planning/management skills
Giri	2017	UK	Leadership in Health Services	Faculty of Occupational Medicine of the Royal College of Physicians	Occupational Health Physician	249	40-60 years	Quantitative	Self-dermined	1-15 years	NHS England Leadership Academy Health-Care Leadership Framework that contains 7 domain: Demonstrating personal qualities, working with others, managing service, improving	Personal Qualities and Improving service	Setting direction, creating the vision, and delivering strategy	Inadequate Leadership development training	The study posit that occupational Health Physician need training in all leadership domains, particularly in managing service and to delivering strategy

				of the UK							service, setting directions, creating vision, and delivery strategy.				
Jalghef	2023	Jordan	Research Square	Jordanian governmental hospitals	Nurse managers	132	35	Quantitative	self perceived competency	<5 years	Hennessy Hicks Training Needs Questionnaire	Getting on with your colleagues, Appraising your own performance,	NR	Inadequate Leadership development training	Working as a member of a team, Providing performance feedback to staff, Providing feedback to colleagues, communication/teamwork,
Gulati	2022	India	Leadership in Health Services	Indian healthcare	Senior health leaders, majorly doctors	416	>40 years	Mixed method	self perceived competency	10-20 years	American College of Healthcare Executives Competency Assessment Tool (2020): communication and relationship management, leadership, professionalism, knowledge of the health-care environment, and business skills and knowledge	NA	NA	leadership skills, time management and communication with patients and other stakeholders	In absence of the leadership training, senior health professionals particularly doctors in India, suffer leadership challenges. Therefore, study suggest efforts to be channeled towards developing these competency
Pillay	2011	South Africa	Public Health Nursing	South African public	Senior nursing managers	94	35-50	Quantitative	Self-assessed proficiency	<5>10years	51 competency Items	Self-management, planning,	NR	Inadequate leadership development training	Legal and ethical issues, organizing, controlling and leading were the identified

				sector hospitals								controlling, and leading			leadership competency gap that needs training development
Xu	2022	China	Journal of Nursing Management	Six large-scale tertiary grade A hospitals in Shanghai, China	clinical nurse managers	27	40 - 50	Qualitative	self perceived competency	<5>10years	Clinical nurse leader competency matrix: Nursing leadership, clinical outcome management, and care environment management	NR	NR	Inadequate leadership training	Nursing leadership (horizontal leadership, team coordination and collaboration with interpersonal communication being highest); clinical outcome management (care quality improvement/patients safety management, knowledge management, and evidence based practices); care environment management (healthcare finance and economic, healthcare system and organization)
Dawson	2013	Australia	Australian Health Review	Regional health service and providing	Allied health professionals	40	NR	Mixed method	Self-rating	NR	NR	NR	NR	Avoidance of addressing personal issues	Empowerment through education, resources development, streamlined documentation and use of best practice protocols

				clinical supervision											
Isibor	2020	Nigeria	European Journal of Investigation in Health Psychology in Education	Nigerian teaching hospitals	Early-career doctors	488	33.5 (±5.7)	Mixed-method	Self-rating	3.3 (±2.7)	NR	NR	NR	Lack of training on leadership skills; Lack of confidence, lack of support from fellow trainees, lack of support from senior doctor, Lack of understanding from other members of the management team; Lack of support from management.	The study concluded that there is a need for a structured leadership skill acquisition programme for ECDs in Nigeria. (communication skills, listening skills, decision making skills, integrity, and being unbiased)
Hartviksen	2020	Norway	Leadership in Health Services	Publicly-financed nursing	Healthcare middle managers’	25	32-57	Qualitative	Self-rating	2-8 years	NR	NR	NR	Grasping complexity (Supervising a complex context Continuousl	Study identified leadership training needs

				homes in Norway Municipality										y developing and compensating) in a conflicting practice (Lacking supported development Striving to meet unclear framework) (continuous knowledge development and compensating contrasted by resource shortages, role conflicts and the lack of trust and cooperation)	
Furunes	2018	Norway	Journal of Clinical Nursing	Community home care setting	Clinical nurse leaders	12	30-39	Qualitative content analysis	Self-rating	5 Years	NR	NR	NR	Autonomy, role clarity, job demands, participating in decision making, skills and competence developmen	Areas of developmental needs include; open communication, attentiveness, taking action, and catering for meaningfulness

														t, and social support.	
Dopelt	2021	Israel	Leadership in Health Services	Israeli health-care system	Physicians in senior management position	13	NR	Exploratory qualitative research	Self-rating	NR	NR	NR	NR	Medical education does not include leadership training	Study identify need for clinicians to undergo appropriate training in management, leadership and interdisciplinary collaboration. Because, leaders should have humane qualities (such as altruism, compassion), be charismatic, inspire and motivate people, have a vision, work collaboratively and be role models for the teams working under them
McGowan	2017	Ireland	PHYSIOTHERAPY THEORY AND PRACTICE	Ireland Healthcare	Physiotherapists	525	NR	Cross-sectional	Self-rating	NR	NR	High perception of leadership competence by physiotherapists who have had leadership training while Communication and professionalism were the most highly rated	Social dominance, Self-awareness, Business acumen	Inadequate leadership training	Study identify need for leadership training to support physiotherapists to assume leadership roles both clinically and non clinically

Ylitalo	2022	Finland	Leadership in Health Services	Finnish primary health-care	Clinical, nursing and medical managers	21	NR	Qualitative	Self-rating	NR	Management competency assessment program as a framework: Evidence, Resources, Knowledge, communication, leadership and change	Developing digital solutions and supporting employees	Day-to-day management and communication with employees; management of large entities	Inadequate leadership training	Study mentioned the need for managers' training according to needs in the future, but based on Six core competencies: Evidence, Resources, Knowledge, communication, leadership and change
Kelly	2023	UK	Journal of Advance Nursing	Samples were drawn across UK	Nurse managers	24	40-65 years	Qualitative	Self-rating	NR	NR	NR	NR	Unique but complex challenges in; preparation for the role, length of time in role, role expectations, managing complexity, status, being political and influencing	Study identify need for leadership training that strengthened successful working relationships with other board colleagues, development of political skills and personal status, coaching and mentoring, working within a supportive team culture and having strong professional networks
Al Ansari	2026	Bahrain & Saudi Arabia	International Journal of Medical	Healthcare institutions and	Senior manager, middle manager, and	27	NR	Qualitative	Self-reported perceptions from	NR	Competency based conceptualisation (no explicit	Personality (integrity, organization), interaction (communication,	Strategic competencies emphasized mainly	Role ambiguity; tension between strategic	Competency-based leadership training; structured development programs for

			Education	medical education organisations	frontline healthcare managers				interview data		formal framework)	active listening), managerial skills (time and task management), work ethics (teamwork, role modelling), mental ability (decision-making, problem solving).	by senior managers; less emphasis among frontline managers.	responsibilities and operational management	healthcare middle managers
Patnaik	2026	India	Journal of Marine Medical Society	Healthcare Leadership and administrative settings	Senior healthcare leaders and healthcare professionals with administrative duties including clinical mangers	355	NR	Quantitative	Validated Likert-scale questionnaire assessing importance and self-rated competency	Clinical and administrative experience reported; Higher experience associated with higher competency ratings	National Centre for Healthcare Leadership (NCHL) Competency Model v2.0	Accountability; execution competencies; people management competencies	Transformational competencies; financial management skills; IT management; HR management; communication skills	Significant gaps between perceived importance and self-assessed competition across domains; lower self-ratings among less experienced leaders	Targeted leadership development programs focusing on transformational leadership, financial management, IT skills, HR management, and communication
Rosser	2025	USA	International	Global	Early-career	46 pre-	NR	Mixed-methods	Online survey	NR	Leadership mentoring and	Leadership confidence;	Uneven mentoring	Time constraints;	Structured mentoring

			Nursing Review	nursing leadership mentoring programme	and established nurse leaders	survey; 32 post-survey					global leadership development orientation	leadership self-efficiency; cultural competence; global leadership awareness; peer learning and reflective leadership development	engagement among some participants	workload pressures; language barriers; inconsistent engagement between mentors and mentees	programmes; facilitator-led peer learning groups; cross-cultural leadership training; sustained mentoring networks
Gaudet	2025	Canda	Occupational Therapy in Health Care	Public and private healthcare organisations	Occupational therapist working as healthcare mangers	27	NR	Qualitative	Interview-based qualitative data analysis	NR	Quadripartite Ethical Framework; Husserlian phenomenological approach	Leadership motivation linked to curiosity, desire to influence decisions, commitment to service improvement, interpersonal leadership and team support	Leadership ideals constrained by organizational structures and performance pressures	Human resource conflicts; heavy workload; staff shortages; turnover; ethical distress; tension between professional values and managerial demands	Leadership preparation addressing ethical decision making, workload management and organizational support for clinical-managers
Hodza-Beganovic	2025	Bosnia and Herzegovina	Human Resources for Health	University hospital clinics in Balkan healthcare	Physicians and nurses in formal healthcare leadership roles	61	NR	Qualitative	Qualitative multi-source data including discussions, interviews,	NR	Leadership as relational and developmental process linked to non-technical skills frameworks	Role clarity and responsibility definition; communication; teamwork; participatory leadership practices; readiness for change	Limited formal leadership training and underdeveloped leadership preparation structures	Hierarchical culture; resistance to change; role ambiguity; dominance of physician leadership roles	Leadership development through mentorship, experiential learning, reflective practice and structured leadership training

				systems					written reflections and field notes						
Ibrahim	2025	Lebanon	British journal of healthcare Management	Private hospitals in Beirut	Physician leaders	100	Majority <40	Quantitative	HEAL leadership competency self-assessment tool	NR	Healthcare Excellence through Advanced Leadership (HEAL) framework	Integrity, selfless leadership, engaging others, critical thinking (align with Personal Qualities and Working with Others)	System transformation and organizational leadership competencies less developed (align with Improving Services and Setting Direction)	Time pressure; financial constraints; negative attitudes from senior physicians; lack of business training	Continuous professional development programs and leadership training including management and business competencies
Van Der Berg-Cloete	2025	South Africa	African journal of health professions education	Public health sector leadership programme	Public health leaders participated in leadership fellowship plus 360° assessors	171	Majority <40	Mixed methods	Questionnaires, reflective material and thematic content analysis	Senior managerial experience common	Leadership development program evaluation framework	Increased leadership confidence, interpersonal leadership capability and reflective leadership awareness (align with Personal Qualities and Working with Others)	Structural system leadership capacity constrained by governance, policy implementation and organizational authority limitations (align with Improving Services	Bureaucracy; limited decision authority; resource shortages; political pressure; organizational resistance to change	Organizational support for leadership practice, mentoring, governance reform and enabling leadership environments

													and Setting Direction)		
Matandela	2024	South Africa	Frontiers in Health Services	Provincial health departments, hospitals, clinics	Nurse leaders across service	153	NR	Qualitative exploratory pilot study using group narrative sessions and semi-structured questionnaires	Qualitative narrative and thematic analysis	NR	Global nursing leadership competency and workforce frameworks (WHO orientation)	Interpersonal leadership support, workforce advocacy, team engagement and professional commitment (aligned with Personal Qualities and Working with Others)	Strategic workforce planning, digital leadership, policy advocacy and career pathway development (align with Improving Services and Setting Direction)	Workforce shortages; migration; workload pressure; weak institutional support; limited leadership preparation	Leadership CPD programmes, workforce planning skills, digital competency development, mentorship and succession planning
Tung	2025	USA	Journal of healthcare leadership	Academic medical centre (Weill Cornell Medicine)	Early-career faculty leaders with academic leadership responsibilities	328	NR	Quantitative	Online Likert-scale surveys assessing leadership skills	Early-career academic leaders	Mayo Leadership Behavior Index (Leader Index)	Professional commitment, interpersonal leadership engagement, feedback and people management skills (aligned with Personal Qualities and Working with Others)	Negotiation skills, managing competing demands, conflict management (align with Improving Services and Setting Direction)	Difficulty managing time, conflict and negotiating responsibilities	Framework-based leadership development programs targeting specific skill gaps
Moreno	2025	USA	Journal of healthcar	Healthcare mana	Healthcare Leaders	96	NR	Quantitative	Online survey combini	Leadership	Kolb's Experiential	Leadership confidence, interpersonal	Rapid decision-making	Crisis pressure, uncertainty	Crisis leadership training, communication

			e management	gement context (Texas)	supervising staff				ng CAT competency tool and C-LEAD self-efficacy scale	experience variable included	Learning Theory	communication, emotional composure during crises (align with Personal Qualities and Working with Others)	under uncertainty and crisis system coordination (align with Improving Services and Setting Direction)	and lack of preparedness for prolonged emergencies	training and simulation-based tabletop drills
Backman	2025	Sweden	Journal of clinical nursing	Residential care facilities for older adults	First-line manager and leaders	34	NR	Quantitative	Web-based Likert-scale relevance survey with I-CVI analysis	NR	Aged-Care Clinical Leadership Qualities Framework (ACLQF) – Person-centred leadership	Person-centred leadership attributes, relational leadership behaviours, ethical leadership orientation (align with Personal Qualities and Working with Others)	Some system-level leadership descriptors rated lower due to limited authority and operational constraints (align with Improving Services and Setting Direction)	Workload pressure, limited decision authority, insufficient organizational support structures	Use of validated leadership framework for leadership training, evaluation and leadership competency development
Pokhrel	2025	Nepal	Asia Pacific Journal of health	Public hospitals	Senior hospital managers	50	NR	Quantitative	MCAP competency assessment	NR	Management Competency Assessment Program (MCAP)	Leading people and organizations; interpersonal communication	Financial/resource management, evidence-	Resource limitations, political complexity, systemic	Scenario-based training, mentorship programmes, case-based leadership

			Management						questionnaire			and relationship management (align with Personal Qualities and Working with Others)	informed decision making, healthcare system knowledge, political acumen and change management (align with Improving Services and Setting Direction)	constraints within public hospitals	education and policy literacy development
Kämäräinen	2025	Finland	Journal of Advanced Nursing	Public healthcare organisations	Nurse leaders	21	NR	Qualitative	Interview-based analysis	Avg 15 years	NR	Interpersonal communication, reflexivity, feedback use, self-awareness, relational leadership (align with Personal Qualities & Working with Others)	Strategic/system leadership not explicitly developed (align with Setting Direction)	Limited feedback; training variability	Communication training; mentoring; reflective practice
Hussein	2025	Iran	Health Promotion Perspectives	Hospitals and healthcare centres	Healthcare manager (hospital, nursing, public health	17	NR	Qualitative	Interview-based qualitative coding	Minimum 1 year	Conceptualised critical thinking as leadership competency	Strategic organizational awareness (understanding culture, mission, political context), adaptive	Limitations in applying critical thinking linked to contextual and	Political influence on decision-making; resource constraints; organizational	Development of critical thinking through training, reflective practice, continuous professional learning, and

					mangers)							leadership (empathy, flexibility, communication, staff-centred management), structured decision-making (problem prioritization, evaluation of alternatives), operational oversight and collaboration (teamwork, supervision), learning and professional development (staff empowerment, continuous learning) align with Personal Qualities & Working with Others	structural constraints (e.g., political environment, organizational complexity) align with Improving Services & Setting Direction	complexity; contextual pressures shaping leadership behaviour	organizational learning culture
Gunter	2025	Australia	Journal of healthcare leadership	Healthcare organisations	Healthcare managers, leaders, and supervisors (multi-professi	276	Mean 45.2	Mixed methods	Likert scale (0-10 ratings) and open-ended qualitative	NR	NR	Ethical leadership, transparency, accountability, inclusive leadership, innovation, resilience, operational balancing	Low perceived sustainability (mean 5.0/10) and moderate support (5.5/10); Difficulty	Staff retention and recruitment issues; time constraints; heavy operational workload limitation	Mentoring and coaching; peer networking; leadership development programs tailored to healthcare; protected time for leadership work; funding for

					onal: nursing, allied health, medical)				responses			demands with long-term vision, mentoring and succession thinking (align with Personal Qualities & Working with Others, with elements of Setting Direction)	sustaining long-term strategic leadership due to operational pressures (align with Improving Services & Setting Direction)	strategic thinking; hierarchical structures restricting innovation; budget constraints; political and financial pressures; performance systems focused on KPIs/finance rather than culture	development; culture-focused performance measures; succession planning
Shikama	2024	Japan	BMC medical education	Clinical healthcare teams (hospital settings)	Physicians involved in clinical leadership within teams	26	NR	Mixed methods	Likert scale (Delphi rounds) and open-ended qualitative response	>10 years clinical experience	CLCF (UK)	Humility, self-discipline, attentive listening, supporting team members, guiding members, fostering psychological safety, promoting team unity; emphasis on relational harmony, conflict avoidance, and team comfort (align strongly with Personal Qualities &	Reduced emphasis on formal system-level leadership tasks (e.g., auditing, resource optimization, formal improvement methodologies); less focus on assertive/individual leadership decision-making	Hierarchical culture limit open expression; risk of groupthink; cultural expectations influencing leadership behaviour; Implicit constraints on assertiveness and decision authority	Development of psychological safety, communication skills, conflict management, delegation, culturally sensitive leadership training, and team-based leadership development approaches

												Working with Others)	(align with Improving Services & Setting Direction)		
Iblasi	2024	Jordan	Nurse media journal of nursing	Public hospitals	First-line manager	130	Mean 37.8 years	Quantitative	FLNM competency scale	Mean 11 years nursing experiences; 7 years managerial experience	FLNM competency scale	Leadership, self-management, staffing and professional development, quality care improvement, spiritual nursing care, and interpersonal managerial competence were emphasized as core areas of competence; female managers and those with recent leadership training reported significantly higher competence (align mainly with Personal Qualities and Working with Others, with some elements of Improving Services)	Financial management and informatics were comparatively more technical/system-oriented domains within the competency structure and representation weaker or less relational areas compared with people-focused domains (align more closely with Improving Services and	Competence was not explained by age or experience alone; demographic context and disparities were evident, including gender differences and effects of social status; cross-sectional self-report limits deeper interpretation of practical leadership barriers	Leadership training was the strongest predictor of higher competence; findings support ongoing structured leadership development, especially training-based support for first-line nurse managers

													Setting Direction)		
Gulati	2024	India	BMJ Lead	Tertiary hospitals	Consultant-level physicians in leadership roles	30	34-65	Qualitative	Interview-based qualitative data	5-35 years	NR	Leadership associated with clinical credibility, experience, multitasking ability, and commitment; some participants viewed women as highly capable and effective (e.g., multitasking, dedication), and leadership seen as achievable through individual effort (align with Personal Qualities & Working with Others)	Perceived lower recognition and access to leadership for women despite capability; stereotypes about women’s effectiveness in certain specialties (e.g., surgery); Leadership opportunities constrained rather than competencies absent (align with Setting Direction & Improving Services)	Specialty-level bias (gendered disciplines, patient distrust of women surgeons); organizational barriers (lack of flexibility, childcare stigma, double standards, age bias); socio-cultural barriers (patriarchy, domestic expectations, motherhood burden, gender conditioning); informal male-dominated power networks explicitly described by	Early integration of leadership training in medical education; continuous leadership development; flexible organizational policies; stronger institutional and family support systems; implementation of equality, diversity, and inclusion (EDI) policies; safe reporting mechanisms

														participants as an “old boys’ club”; Expectation for women to adopt more aggressive behaviours to be taken seriously	

Supplementary Table S4. Mapping of reported leadership comptencies from included studies to the domains of the CLCF

Study	Reported competencies (as in study)	Mapped CLCF Domains
Liang (2020)	Low: conflict management; performance management and improvement; new skills development; making decision; making change	**Working with Others:** conflict management<br>**Managing Services:** performance management and improvement<br>**Demonstrating Personal Qualities:** new skills development<br>**Setting Direction**: making decision<br>**Improving Services:** making changes
Ireri (2011)	High: personal qualities; working with others; managing service<br>Low: service improvement; working with others; self-management	**Demonstrating Personal Qualities**: personal qualities; self-management<br>**Working with Others:** working with others<br>**Managing Services:** managing service<br>**Improving Services:** service improvement

Ireri (2017)	High: Managing services; working with others; personal qualities<br>Low: Improving services; setting direction	**Managing Services:** Managing services<br>**Working with Others:** working with others<br>**Demonstrating Personal Qualities:** personal qualities<br>**Improving Services:** Improving services<br>**Setting Direction:** setting direction
Mai (2021)	Competencies: effective and timely communication; teaching strategies in the clinical setting; effective time management and resource utilization; Self-care and emotional regulation techniques to build resilience	**Working with Others:** effective and timely communication<br>**Improving Services:** teaching strategies in the clinical setting<br>**Managing Services:** effective time management and resource utilization<br>**Demonstrating Personal Qualities:** self-care and emotional regulation techniques to build resilience
Fraser (2015)	Competencies: leading a team; innovative problem solving; coaching and developing others; resolving conflict; time management; self management; Embracing change; Self-awareness	**Working with Others:** leading a team; coaching and developing others; resolving interpersonal conflict<br>**Improving Services:** innovative problem solving; Embracing change<br>**Managing Services:** time management<br>**Demonstrating Personal Qualities:** self management; Self-awareness
Gulati (2021)	Low: Setting directions; Working with others; Managing services; Knowledge of HR, procurement, financial and contracts management	**Setting Direction:** Setting directions<br>**Working with Others:** Working with others<br>**Managing Services:** Managing services; Knowledge of HR, procurement, financial and contracts management
Fiest (2017)	Competencies: communication; inspiration/motivation; conflict resolution; relationship building; self awareness; strategic planning; policy and program	**Working with Others:** communication; inspiration/motivation; conflict resolution; relationship building; teambuilding; Supervision and mentoring<br>**Demonstrating Personal Qualities:** self awareness

	development; team building; change theory; Recruitment and retention; human resources policies and procedures; regulatory compliance; financial and budgetary planning; supervision and mentoring; quality improvement	**Improving Services:** strategic planning; policy and program development; change theory; quality improvement<br>**Managing Services:** Recruitment and retention; human resources policies and procedures; regulatory compliance; financial and budgetary planning
Liang (2013)	Competencies: Knowledge of health care environment and the organization; Interpersonal communication qualities and relationship management; Leading and managing change; Evidence informed decision-making; communication qualities; Self-management and resilience; Operations, administration and resource management	**Managing Services:** Knowledge of health care environment and the organization; Operations, administration and resource management<br>**Working with Others:** Interpersonal communication qualities and relationship management; communication qualities<br>**Improving Services:** Leading and managing change<br>Setting Direction: Evidence informed decision-making<br>**Demonstrating Personal Qualities:** Self-management and resilience
Mrayyan (2022)	High: Leadership and clinical practice<br>Low: financial and service management	**Working with Others:** Leadership and clinical practice<br>**Managing Services:** financial and service management
Warshawsky (2019)	High: human resources management; clinical practices; diversity management; technology<br>Low: financial management; strategic management; performance	**Managing Services:** human resources management; financial management; clinical practices; technology<br>**Working with Others:** diversity management<br>**Improving Services:** performance improvement<br>**Setting Direction:** strategic management; foundational thinking

	improvement; foundational thinking	
Liang (2020b)	High: Evidence; Knowledge; communication; leadership<br>Low: Resources; Change; Conflict management; innovative team work; performance	**Working with Others:** communication; leadership; Conflict management; innovative teamwork<br>**Managing Services:** Resources; performance; Knowledge<br>**Improving Services:** Change
Fanelli (2022)	High: Problem solving; planning; operation and project management; leadership and internal communication; quality improvement<br>Low: financial management; Human resource management; organizational design; External communication; output and outcome assessment	**Working with Others:** leadership and internal communication; External communication<br>**Managing Services:** operation and project management; financial management; Human resource management<br>**Improving Services:** quality improvement; Problem solving<br>**Setting Direction:** planning; organizational design; output and outcome assessment
Dickinson (2013)	Not explicitly reported	-
Gulati (2019)	High: personal Qualities; improving service<br>Low: working with others; managing service; setting direction; knowledge of HR, procurement, finance, contract management; ability	**Demonstrating Personal Qualities:** Personal Qualities; time and stress management<br>**Working with Others:** Working with others<br>**Managing Services:** managing service; knowledge of HR, procurement, finance, contract management<br>**Improving Services:** Improving service; conducting need analysis

	to influence decision; time and stress management; conducting need analysis; identify and prioritize requirement	**Setting Direction:** setting direction; ability to influence decision; identify and prioritize requirement
Welch (2021)	High: Nurse with >5 years of leadership experience were more competent<br>Low: knowledge of quality improvement strategies; ability to lead multi-generational work teams (communication style; motivation factors; conflict management; work expectations)	**Improving Services:** knowledge of quality improvement strategies<br>**Working with Others:** ability to lead multi-generational work teams; communication style; motivation factors; conflict management
Miltner (2015)	Competencies: Managing Versus Leading; Gaining a Voice; Garnering Support	**Managing Services:** Managing Versus Leading<br>**Working with Others:** Gaining a Voice; Garnering Support
Liou (2021)	Competencies: Leadership (advocacy; strategic thinking; establishing policy; system and structure; being politically astute; external awareness; leading change; leading on vision and values; global perspective and mindset); Business literacy (performance improvement; financial management; information management and technology; evidence	**Demonstrating Personal Qualities:** global perspective and mindset; analytic thinking<br>**Working with Others:** effective communication; relationship building and management; interpersonal skills; influencing and negotiation<br>**Managing Services:** performance improvement; financial management; information management and technology; Planning and organization; execution<br>**Improving Services:** leading change; creativity and innovation; quality and safety orientation; problem solving<br>**Setting Direction:** strategic thinking; establishing policy; system and structure; external awareness; evidence based decision making skills; setting direction

	based decision making skills; creativity and innovation; setting direction); Integrative skills (Planning and organization; quality and safety orientation; execution; problem solving; analytic thinking); Communication and relationship (effective communication; relationship building and management; interpersonal skills; influencing and negotiation)	
Bigbee (2010)	High: communication; cultural competency; leadership skills<br>Low: Policy development/program planning; analytic assessment; financial planning/management	**Working with Others:** communication; cultural competency; leadership skills<br>**Setting Direction:** Policy development/program planning; analytic assessment<br>**Managing Services:** financial planning/management
Giri (2017)	High: Personal Qualities; Improving service<br>Low: Setting direction; creating the vision; delivering strategy	**Demonstrating Personal Qualities:** Personal Qualities<br>**Improving Services:** Improving service<br>**Setting Direction:** Setting direction; creating the vision; delivering strategy
Jalghef (2023)	High: Getting on with your colleagues; Appraising your own performance	**Working with Others:** Getting on with your colleagues<br>**Demonstrating Personal Qualities:** Appraising your own performance
Gulati (2022)	Competencies: leadership skills; time management; Communication with patients and other stakeholders	**Working with Others:** communication with patients and other stakeholders; leadership skills<br>**Managing Services:** time management

Pillay (2011)	High: Self-management; planning; controlling; leading	**Demonstrating Personal Qualities:** Self-management<br>**Managing Services:** planning; controlling<br>**Working with Others:** leading
Xu (2022)	Competencies: Nursing leadership; clinical outcome management; care environment management	**Working with Others:** Nursing leadership<br>**Improving Services:** clinical outcome management<br>**Managing Services:** care environment management
Dawson (2013)	Not explicitly reported	-
Isibor (2020)	Competencies: communication skills; listening skills; decision making skills; integrity; being unbiased	**Working with Others:** communication skills; listening skills<br>**Setting Direction:** decision making skills<br>**Demonstrating Personal Qualities:** integrity; being unbiased
Hartviksen (2020)	Competencies: Supervising a complex context; Continuously developing and compensating; Lacking supported development; Striving to meet clear framework; continuous knowledge development and compensating; resource shortages; role conflicts; lack of trust and cooperation	**Managing Services:** Supervising a complex context; resource shortages; role conflicts<br>**Demonstrating Personal Qualities:** Continuously developing and compensating; Continuous knowledge development and compensating<br>**Setting Direction:** Striving to meet clear framework<br>**Working with Others:** lack of trust and cooperation
Furunes (2018)	Competencies: Autonomy; role clarity; job demands; participating in decision making; skills and competence	**Demonstrating Personal Qualities:** Autonomy; skills and competence development<br>**Setting Direction:** participated in decision making<br>**Managing Services:** role clarity; job demands

	development; social support	**Working with Others:** social support
Dopelt (2021)	Competencies: humane qualities (altruism; compassion); charismatic; inspire and motivate people; have a vision; work collaboratively; role models	**Demonstrating Personal Qualities:** humane qualities (altruism; compassion); charismatic<br>**Working with Others:** inspire and motivate people; work collaboratively; role models<br>**Setting Direction:** have a vision
McGowan (2017)	High: Communication; professionalism<br>Low: social dominance; Self-awareness; Business acumen	**Working with Others:** Communication<br>**Demonstrating Personal Qualities:** professionalism; Self-awareness<br>**Setting Direction:** Business acumen
Ylitalo (2022)	High: Developing digital solutions; supporting employees<br>Low: Day-to-day management and communication with employees; management of large entities	**Improving Services:** Developing digital solutions<br>**Working with Others:** supporting employees; communication with employees<br>**Managing Services:** Day-to-day management; management of large entities
Kelly (2023)	Competencies: preparation for the role; length of time in role; role expectations; managing complexity; status; being political and influencing	**Managing Services:** managing complexity<br>**Setting Direction:** being political and influencing; role expectations<br>**Demonstrating Personal Qualities:** preparation for the role<br>**Working with Others:** status (the ability to recognize and navigate the professional power dynamics, hierarchies, and levels of influence between yourself and others to ensure effective collaboration)
Alansari (2026)	Competencies: Personality (integrity; organization); interaction (communication;	**Demonstrating Personal Qualities:** Personality (integrity; organization)<br>**Working with Others:** interaction (communication; active listening); teamwork; role modeling

	active listening); managerial skills (time and task management); work ethics (teamwork; role modeling); mental ability (decision-making; problem solving)	**Managing Services:** managerial skills (time and task management)<br>**Setting Direction:** decision-making; problem solving
Patnaik (2026)	High: Accountability; execution competencies; people management competencies<br>Low: Transformational competencies; financial management skills; IT management; HR management; communication skills	**Managing Services:** execution competencies; financial management; IT management; HR management<br>**Working with Others:** people management competencies; communication skills<br>**Demonstrating Personal Qualities:** Accountability<br>**Setting Direction:** Transformational competencies
Rosser (2025)	Competencies: Leadership confidence; leadership self-efficiency; cultural competence; global leadership awareness; peer learning; reflective leadership development	**Demonstrating Personal Qualities:** Leadership confidence; self-efficiency; reflective leadership development<br>**Working with Others:** cultural competence; peer learning<br>**Setting Direction:** global leadership awareness
Gaudet (2025)	Competencies: leadership motivation; curiosity; influence decisions; commitment to service improvement; interpersonal leadership; team support	**Demonstrating Personal Qualities:** curiosity; leadership motivation<br>**Working with Others:** interpersonal leadership; team support<br>**Improving Services:** commitment to service improvement<br>**Setting Direction:** influence decisions

Hodza-Beganovic (2025)	Competencies: role clarity; communication; teamwork; participatory leadership; readiness for change	**Managing Services:** role clarity<br>**Working with Others:** communication; teamwork; participatory leadership<br>**Improving Services:** readiness for change
Ibrahim (2025)	High: Integrity; selfless leadership; engaging others; critical thinking<br>Low: system transformation; organizational leadership competencies	**Demonstrating Personal Qualities:** Integrity; selfless leadership; critical thinking<br>**Working with Others:** engaging others<br>**Setting Direction:** system transformation; organizational leadership competencies
Van Der Berg-Cloete (2025)	High: leadership confidence; interpersonal leadership capability; reflective leadership awareness<br>Low: system leadership capacity; governance; policy; implementation organizational authority	**Demonstrating Personal Qualities:** leadership confidence; reflective leadership awareness<br>**Working with Others:** interpersonal leadership capability<br>**Setting Direction:** system leadership capacity; governance; policy; implementation organizational authority
Matandela (2024)	Competencies: interpersonal leadership support; workforce advocacy; team engagement; professional commitment; strategic workforce planning; digital leadership; policy advocacy; career path development	**Working with Others:** interpersonal leadership support; team engagement<br>**Demonstrating Personal Qualities:** professional commitment<br>**Setting Direction:** strategic workforce planning; policy advocacy; career path development<br>**Improving Services:** digital leadership
Tung (2025)	Competencies: professional commitment; interpersonal leadership	**Demonstrating Personal Qualities:** professional commitment<br>**Working with Others:** interpersonal leadership engagement; feedback; people management skills; conflict management

	engagement; feedback; people management skills; negotiation skills; managing competing demands; conflict management	**Setting Direction:** negotiation skills; managing competing demands
Moreno (2025)	Competencies: leadership confidence; interpersonal communication; emotional composure; decision-making; crisis system coordination	**Demonstrating Personal Qualities:** leadership confidence; emotional composure<br>**Working with Others:** interpersonal communication<br>**Setting Direction:** decision-making; crisis system coordination
Backman (2025)	Competencies: person-centred leadership; relational leadership behaviours; ethical leadership orientation	**Demonstrating Personal Qualities:** ethical leadership orientation<br>**Working with Others:** person-centred leadership; relational leadership behaviours
Pokhrel (2025)	Competencies: Leading people and organizations; interpersonal communication; relationship management; financial/resource management; evidence-informed decision making; healthcare system knowledge; political acumen; change management	**Working with Others:** interpersonal communication; relationship management; leading people<br>**Managing Services:** financial/resource management; healthcare system knowledge<br>**Setting Direction**: evidence-informed decision making; political acumen<br>**Improving Services:** change management
Kämäräinen (2025)	Competencies: interpersonal communication; reflexivity; feedback use; self-awareness; relational leadership	**Working with Others:** interpersonal communication; relational leadership; feedback use<br>**Demonstrating Personal Qualities:** reflexivity; self-awareness

Hussein (2025)	Competencies: strategic organizational awareness; adaptive leadership; structured decision-making; operational oversight; teamwork; supervision; learning and professional development	**Setting Direction:** strategic organizational awareness; structured decision-making<br>**Working with Others:** teamwork; supervision<br>**Managing Services:** operational oversight<br>**Demonstrating Personal** Qualities: adaptive leadership; learning and professional development
Gunter (2025)	Competencies: ethical leadership; transparency; accountability; inclusive leadership; innovation; resilience; mentoring; succession thinking	**Demonstrating Personal** Qualities: ethical leadership; transparency; accountability; resilience<br>**Working with Others:** inclusive leadership; mentoring<br>**Improving Services:** innovation<br>**Setting Direction:** succession thinking
Shikama (2024)	Competencies: humility; self-discipline; attentive listening; supporting team members; guiding members; fostering psychological safety; promoting team unity	**Demonstrating Personal Qualities:** humility; self-discipline<br>**Working with Others:** attentive listening; supporting team members; guiding members; fostering psychological safety; promoting team unity
Iblasi (2024)	Competencies: leadership; self-management; staffing and professional development; quality care improvement; spiritual nursing care; interpersonal managerial competence	**Demonstrating Personal Qualities:** self-management<br>**Working with Others:** interpersonal managerial competence<br>**Managing Services:** staffing and professional development<br>**Improving Services:** quality care improvement<br>**Setting Direction:** leadership
Gulati (2024)	Competencies: clinical credibility; experience; multitasking ability; commitment	**Demonstrating Personal Qualities:** commitment; multitasking ability<br>**Working with Others:** clinical credibility<br>**Managing Services:** experience

Note: mapping was based on the competency constructs extracted from each study and aligned conceptually to the most relevant CLCF domains. Some studies contributed to more than on domain.
